# Atherosclerotic Pre-Conditioning Affects the Paracrine Role of Circulating Angiogenic Cells Ex-Vivo

**DOI:** 10.3390/ijms21155256

**Published:** 2020-07-24

**Authors:** Sara Eslava-Alcon, Mª Jesús Extremera-García, Ismael Sanchez-Gomar, Lucía Beltrán-Camacho, Antonio Rosal-Vela, Javier Muñoz, Nuria Ibarz, Jose Angel Alonso-Piñero, Marta Rojas-Torres, Margarita Jiménez-Palomares, Almudena González-Rovira, Rosario Conejero, Esther Doiz, Manuel Rodriguez-Piñero, Rafael Moreno-Luna, Mª Carmen Durán-Ruiz

**Affiliations:** 1 Biomedicine, Biotechnology and Public Health Department, Cádiz University, 11002 Cádiz, Spain; sara.eslava@gm.uca.es (S.E.-A.); maria.jesus.extremera.garcia@gmail.com (M.J.E.-G.); ismael.sanchez@uca.es (I.S.-G.); lucia.beltrancamacho@alum.uca.es (L.B.-C.); antonio.rosal@inibica.es (A.R.-V.); joseangel.alonsopi@alum.uca.es (J.A.A.-P.); marta.rojas@gm.uca.es (M.R.-T.); margarita.jimenezpalomares@uca.es (M.J.-P.); almudena.gonzalez@uca.es (A.G.-R.); 2 Institute of Research and Innovation in Biomedical Sciences Cadiz (INIBICA), 11009 Cádiz, Spain; 3 Proteomics Unit, Spanish National Cancer Research Centre (CNIO), 28029 Madrid, Spain; jmunozpe@cnio.es (J.M.); nibarz@cnio.es (N.I.); 4 ISCIII-ProteoRed, 28029, 28029 Madrid, Spain; 5 Angiology& Vascular Surgery Unit, Hospital Universitario Puerta del Mar, 11009 Cádiz, Spain; rosarioconejero@gmail.com (R.C.); edoiz@comcadiz.com (E.D.); mropinero@gmail.com (M.R.-P.); 6 Laboratory of Neuroinflammation, Hospital Nacional de Paraplejicos, SESCAM, 45071 Toledo, Spain; rmluna@sescam.jccm.es

**Keywords:** circulating angiogenic cells, endothelial colony forming cells, paracrine role, secretome, atherosclerotic factors, angiogenesis, proteomics

## Abstract

In atherosclerosis, circulating angiogenic cells (CAC), also known as early endothelial progenitor cells (eEPC), are thought to participate mainly in a paracrine fashion by promoting the recruitment of other cell populations such as late EPC, or endothelial colony-forming cells (ECFC), to the injured areas. There, ECFC replace the damaged endothelium, promoting neovascularization. However, despite their regenerative role, the number and function of EPC are severely affected under pathological conditions, being essential to further understand how these cells react to such environments in order to implement their use in regenerative cell therapies. Herein, we evaluated the effect of direct incubation ex vivo of healthy CAC with the secretome of atherosclerotic arteries. By using a quantitative proteomics approach, 194 altered proteins were identified in the secretome of pre-conditioned CAC, many of them related to inhibition of angiogenesis (e.g., endostatin, thrombospondin-1, fibulins) and cell migration. Functional assays corroborated that healthy CAC released factors enhanced ECFC angiogenesis, but, after atherosclerotic pre-conditioning, the secretome of pre-stimulated CAC negatively affected ECFC migration, as well as their ability to form tubules on a basement membrane matrix assay. Overall, we have shown here, for the first time, the effect of atherosclerotic factors over the paracrine role of CAC ex vivo. The increased release of angiogenic inhibitors by CAC in response to atherosclerotic factors induced an angiogenic switch, by blocking ECFC ability to form tubules in response to pre-conditioned CAC. Thus, we confirmed here that the angiogenic role of CAC is highly affected by the atherosclerotic environment.

## 1. Introduction

The potential use of endothelial progenitor cells (EPC) for cell therapy in cardiovascular diseases (CVD) has been largely evaluated due to their assigned role in vascular repairing and tissue revascularization [1,2,3]. In atherosclerosis itself, EPC seem to play a pivotal role in atherogenesis and arterial healing after injury [4,5,6]. Briefly, EPC become activated in response to endothelial damage, mobilize to the arterial thrombi, where they initiate an endogenous response to restore and replace the dysfunctional endothelium [4]. Since their discovery in 1997 [7], many researchers have focused on clarifying the identity of these cells, their nature, and characteristics. To date, two main sub-populations have been formally defined from the initially named EPC [8,9,10]: early EPC (eEPC), also called circulating angiogenic cells (CAC) or myeloid angiogenic cells (MAC), with limited proliferation capacity (lasting 7–10 days after isolation) and angiogenic potential through paracrine mechanisms [11,12]; and late outgrowth cells or endothelial colony-forming cells (ECFC), highly proliferative and with enormous vasculogenic properties, probably by direct engraftment and/or replacement of damaged cells [8,9].

Both sets of cells have been used alone or combined in diverse pre-clinical or clinical studies, with certain success in the restoration, among others, of ischemic tissues such as in critical limb ischemia [1,2,13,14,15,16]. Moreover, mixed transplantation seems to promote the revascularization effect [13], since these cells participate in a coordinated manner [10]: CAC are recruited to the damaged tissue and secrete paracrine factors that promote the migration of ECFC to the injured site to restore the integrity of the vascular wall. Unfortunately, diverse CVD risk factors impair the number of EPC, thus, limiting their effect in vascular regeneration [17]. Indeed, the number of circulating EPC has become a predicting factor of a patient’s outcome [18,19,20,21]. In atherosclerosis itself, the number of circulating EPC is a strong predictor of atherosclerotic plaque recurrence in the common carotid artery, with decreased number of EPC correlating with the presence and progression of preclinical atherosclerosis [18,22,23].

In this regard, we and other groups have focused on understanding how these cells are affected under pathological conditions, in order to be able to increase their number while keeping and promoting their mobilization and regenerative properties for cell therapy purposes [5,24,25,26]. Several assays have shown that EPC are susceptible to inflammatory and pro-atherogenic factors, promoting an increased neovascularization of ischemic tissues with pre-stimulated cells [23,27]. Very recently, we described an experimental approach based on the incubation ex-vivo of healthy CAC with factors released by atherosclerotic arteries to the circulating media, in an attempt to understand how these cells become activated under such an environment [5]. Remarkably, the atherosclerotic factors promoted an increased mobilization of CAC, which correlated with an increased expression of intracellular proteins related to cell proliferation, migration, and vascular remodeling [5,18]. Herein, we have moved forward, identifying the differential factors released by CAC in response to the atheroma plaque secretome. Our results indicate that, under atherosclerotic conditions, CAC released factors promote a reduction of angiogenesis and migration of ECFC compared to a healthy, non-pathological situation. Thus, we have corroborated the potential use of the healthy CAC secretome itself to promote angiogenesis or ECFC migration ex vivo, as previously seen [13] and, moreover, in presence of atherosclerotic factors angiogenesis can be reverted. The relevance of such an angiogenic switch is discussed.

## 2. Results

### 2.1. The Secretion Protein Profile of CAC Is Affected by the Incubation Ex-Vivo with Atherosclerotic Factors

**Figure 1:**
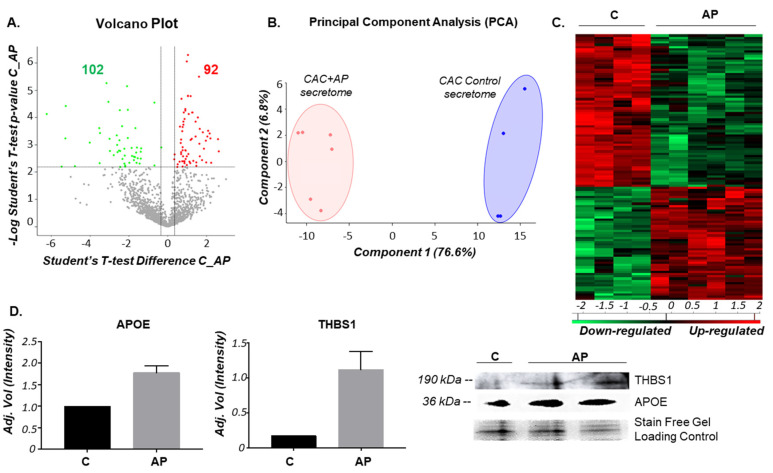
Differential secretion protein profile of circulating angiogenic cells (CAC) in presence/absence of atherosclerotic factors. CAC were incubated in presence/absence (AP/C) of atherosclerotic factors and the secretion protein profile was analyzed by mass spectrometry using a label-free quantitative approach. Results were analyzed with MaxQuant and Perseus software. The figure includes a: (**A**) Volcano plot, which shows up- (red) and down-regulated (green) proteins, using a *p*-value < 0.05 and log_2_ ratio >1.35 or <−1.35 as cut-off values to consider significant changes; (**B**) principal component analysis (PCA) graph, clearly distinguishing between treated (CAC+AP) and untreated CAC (Control) supernatant based on the differential secretion profile; (**C**) hierarchical clustering of the proteins differentially present in the secretomes of untreated (C) and CAC stimulated with the atherosclerotic factors (AP); (**D**) validation by western blot of the proteomic results seen for trombospondin-1 (THBS1) and apolipoprotein E (APOE), both up-regulated in the secretome of CAC+AP stimulated cells.

As a first step, the identity of CAC was confirmed by flow cytometry as previously described [12] (Supplementary Figure S1). In order to identify a differential secretion pattern of CAC in response to an atherosclerotic environment, a label-free quantitative proteomic analysis was carried out comparing the secretome of CAC in basal medium (C) with the secretome of CAC pre-treated ex-vivo with atherosclerotic factors (CAC+AP or AP). Thus, from the total of 1516 proteins identified in the conditioned media of CAC, 92 proteins appeared up-regulated and 102 down-regulated in the secretome of CAC after 2 h incubation ex-vivo with the atherosclerotic released factors (CAC+AP, or AP) compared to the secretome of un-stimulated CAC (C) (Figure 1A). The full list of protein IDs up- and down-regulated is shown in the Supplementary Material (Table S1).

Furthermore, PCA classification analysis (Figure 1B) and hierarchical clustering (Figure 1C) based on differential secretion profiles showed two clear differentiated groups, treated vs. un-treated cells. In addition, validation of proteomic results was done with two proteins, apolipoprotein E (APOE) and trombospondin-1 (THBS1), both of them up-regulated in the secretome of CAC-treated cells (AP) compared to CAC controls (Figure 1D).

**Figure 2:**
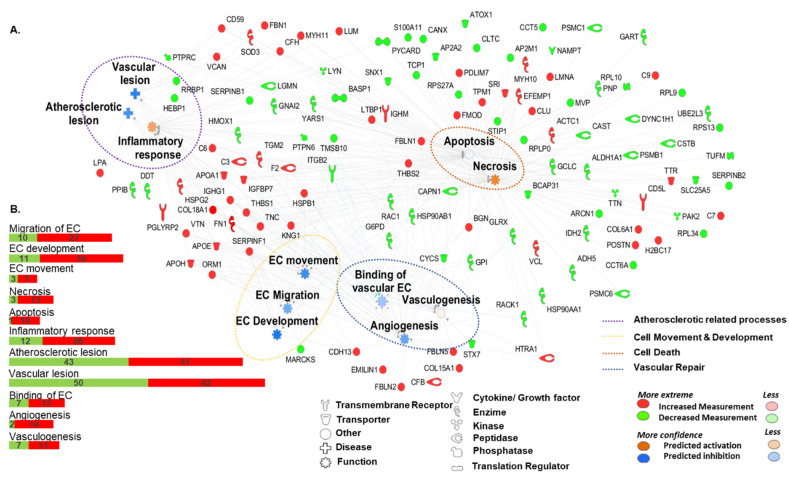
Functional classification of up- and down-regulated proteins in the CAC secretome. (**A**) Functional network based on the information stored in the IPA Software Knowledge database, highlighting major roles in which differential proteins were related: Vascular repair; Atherosclerotic related processes; cell death and cell movement and development. (**B**) Graphical representation with the number of proteins up- (red) or down-regulated (green) in the different functions described. EC: Endothelial cells.

The protein changes detected (Table 1 and Figure 2) in the secretomes of CAC in response to atherosclerotic factors (up- and down-regulated) were mainly correlated, according to IPA, with several functions: Cardiovascular system development [including development of vasculature (48 proteins), vasculogenesis (37), angiogenesis (41), and cell movement (20) among others); *cardiovascular disease* [atherosclerosis (11), peripheral vascular disease (23), or vascular lesion (16)]; hematological system development and function, or inflammatory response. At the cellular level, the changes seen appeared to be related to endothelial cell migration (18), cell death and survival [apoptosis (84) necrosis (92)], or cell to cell signaling interactions [adhesion/binding of blood cells (30)]. Moreover, according to IPA (a software, which based on biomedical literature and integrated databases, allows to determine the most probable pathways or functions in which proteins are involved), protein variations in the secretome of CAC treated with atherosclerotic factors represented an impairment of angiogenesis and migration, as well as apoptosis, but an increase of necrosis (Figure 2A) as well as a promotion of the inflammatory response. Interestingly, an important set of proteins found as up-regulated in the “atherosclerotic” pre-conditioned CAC were extracellular matrix (ECM) related proteins with known anti-angiogenic properties: restin (COL15A1), endostatin (COL18A1), emilin-1 (EMILIN1), thrombospondin 1 and 2 (THBS1, THBS2), fibulins 1, 2, and 5 (FBLN1, FBLN2, FBLN5), heparan sulfate proteoglycan 2 (HSPG2), also known as perlecan, serpin F1 (SERPINF1), fibronectin-1 (FN1), or tenascin (TNC). On the other hand, proteins such as heat shock protein 90 (HSP90) or heme oxygenase-1 (HMOX1) were down-regulated in CAC in response to the AP secretome. Complete and detailed information regarding protein changes can be found in Supplementary Table S2.

### 2.2. CAC Secretomes in Presence/Absence of Atherosclerotic Factors Alter Angiogenesis Potential of ECFC “Ex Vivo”

**Figure 3:**
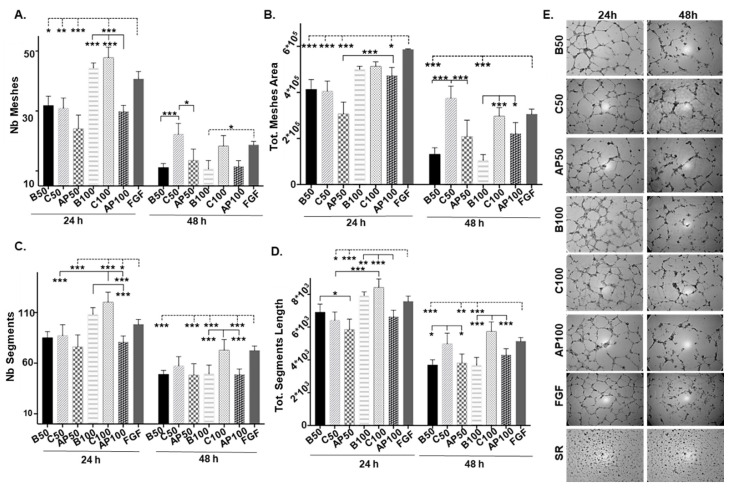
Effect of CAC secretome on endothelial colony-forming cells (ECFC) angiogenic potential. ECFC were treated (n:3 per condition) with either serum free-EBM-2 medium, basal condition (B50, B100); CAC control secretome (C50, C100) or the secretome of CAC pre-incubated with AP factors (AP50, AP100), at different concentrations (50 and 100 ng/μL), for 24 and 48 h. In addition, ECFC were incubated with FGF (angiogenesis activator) and SR (inhibitor) as positive and negative angiogenic controls. Graphical results indicate (**A**) number of meshes, (**B**) total meshes area (pixels^2^), (**C**) number of segments, and (**D**) total segments length (pixels). (**E**) Representative image of all conditions tested at 24 and 48 h are shown. Images were taken with an inverted phase-contrast microscope, under 40× magnification. Data were presented as mean ±SE. The most relevant significant changes are shown (* *p* < 0.05; ** *p* < 0.01; *** *p* < 0.001), while the entire set of *p*-values < 0.05 has been included in Supplementary Table S3. Significant differences between conditions (—); Significant differences vs. the activator (FGF) (----).

Taking into account the proteomic results, we decided to evaluate whether the incubation ex vivo of CAC with the atherosclerotic factors could have indeed an effect over the paracrine role assigned to these cells (Figure 3). Thus, ECFC were incubated with the secretome of control CAC (C, unstimulated) or the secretome of CAC pre-incubated with the AP factors (AP) at different times (24 and 48 h) with two different concentrations of the atherosclerotic factors (50 and 100 ng/μL, AP50 and AP100, respectively). A basal control was used in all assays, incubating ECFC with EBM-2 media without FBS (B, basal media) and equivalent amounts of growth factors to the amounts added of the CAC secretome (B50 and B100), in order to consider exclusively the effect of the CAC secreted factors over the ECFC. Our results indicated that after 24 h, angiogenesis was induced in all cases (compared to baseline), but significant differences were seen in the angiogenic potential of ECFC depending on whether they were incubated with the secretome of CAC pre-stimulated (AP) or not (C) with atherosclerotic factors.

Overall, after 24 h, ECFC incubated with the secretomes of un-stimulated CAC (C) showed similar levels of angiogenesis than un-treated ECFC (Basal, B), with similar number of meshes (Figure 3A) and meshes area (Figure 3B) but also regarding the number (Figure 3C) and length of the segments (Figure 3D), independently of the concentration of CAC secretome used, although the values were slightly higher (not significant) at 100 ng/µL (C100) than 50 ng/µL (C50). Interestingly, the incubation of ECFC with 100 ng/µL of the CAC control secretome (C100) resulted in better values (more meshes and segments) than the activator (FGF) itself (*p*-value < 0.001). On the other hand, ECFC incubated with the secretome of stimulated CAC (AP50, AP100) showed lower number of meshes and segments than basal (B50, B100) and control ECFC (C50, C100), and these differences were significant (*** *p*-value < 0.001, N° meshes and n° segments, ** *p*-value < 0.01 for segment length) at higher concentrations of CAC+AP secretome (100 ng/µL, AP100).

After 48 h, differences were more pronounced. Overall, while angiogenesis decreased in ECFC under basal conditions compared to 24 h values for all parameters (number and area of meshes and also number and length of tubule segments measured), for the ECFC treated with un-stimulated, control CAC, the number and area of meshes as well as the total length were significantly higher at 48 h with both concentrations (C50 and C100) than in basal conditions. On the contrary, angiogenesis was significantly impaired in ECFC incubated with the secretome of CAC+AP (AP50, AP100) compared to ECFC treated with control CAC secretome (less number and area of meshes, or less segment lengths), although these levels were never lower than basal ECFC. Herein at least, the negative effect of the CAC+AP secretome was not dose-dependent, with both conditions (AP50, AP100) showing similar effect. Supplementary Table S3 includes the *p*-values calculated for all significant differences seen between the conditions tested.

### 2.3. CAC Secretomes in Presence/Absence of Atherosclerotic Factors Effect on ECFC Migration “Ex Vivo”

**Figure 4:**
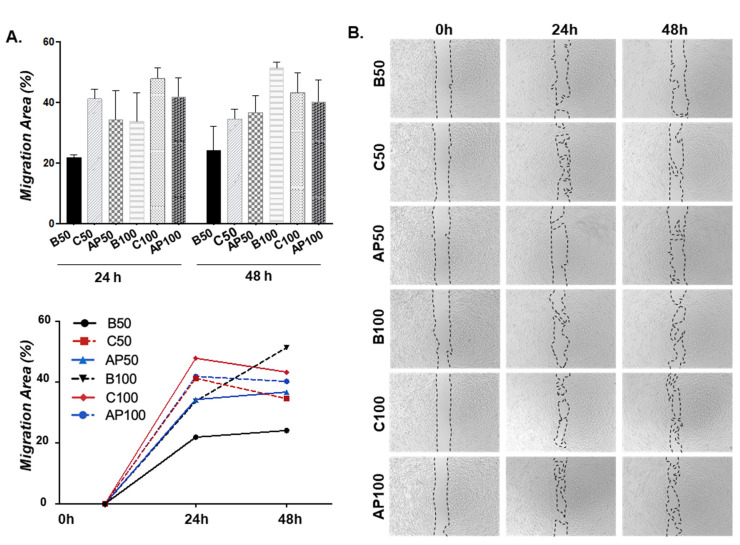
Effect of CAC secretome on ECFC migration. ECFC were treated (n:3 per condition) with either serum free EBM-2 medium as basal condition (B50, B100), CAC control secretome (C50, C100) or the secretome of CAC pre-incubated with AP factors (AP50, AP100) at different concentrations (50 and 100 ng/μL) for 24 and 48 h. (**A**) Graphical representation of the migration area detected per condition, represented as the percentage of migration area (%) calculated vs. the area detected at baseline (time: 0 h). A graphical representation of relative temporal changes is also shown. (**B**) Representative image of migration assays for all conditions tested at baseline (time 0), 24 and 48 h are shown. Images were taken with an inverted phase-contrast microscope, under 40× magnification. Results are presented as mean ± SE.

We next tested the effect of CAC secretomes in basal (C) or atherosclerotic environments (AP) over ECFC migration, by using a wound migration assay (Figure 4). Interestingly, after 24 h ECFC migration increased in response to the C secretome, compared to basal ECFC (B50 and B100) whereas ECFC incubated with the secretome of CAC+AP (AP50 and AP100) showed a lower ability to migrate than C, although, interestingly, more than basal conditions, un-stimulated ECFC (B). Although these changes were not significant, the results indicated that CAC (under basal/unaffected conditions) promoted the migration of ECFC, while this effect was relatively blocked after the incubation of CAC with the atherosclerotic secretomes. After 48 h, migration areas (%) did barely change compared to 24 h, except for AP50, which showed similar migration % than C50, in both cases higher than B50. Also, ECFC stimulated with either control CAC (C100) or CAC+AP secretomes (AP100) migrated in similar levels, and less than basal ECFC (B100).

Overall, migration was mainly promoted after 24 h, with the highest increase seen, compared to basal ECFC, in ECFC stimulated with the secretome of control CAC, while the secretome of CAC+AP seemed to slightly block such migration, but not significantly, since these cells also migrated more than basal, un-treated ECFC.

### 2.4. CAC Secretomes Effect over ECFC Apoptosis “Ex Vivo”

**Figure 5:**
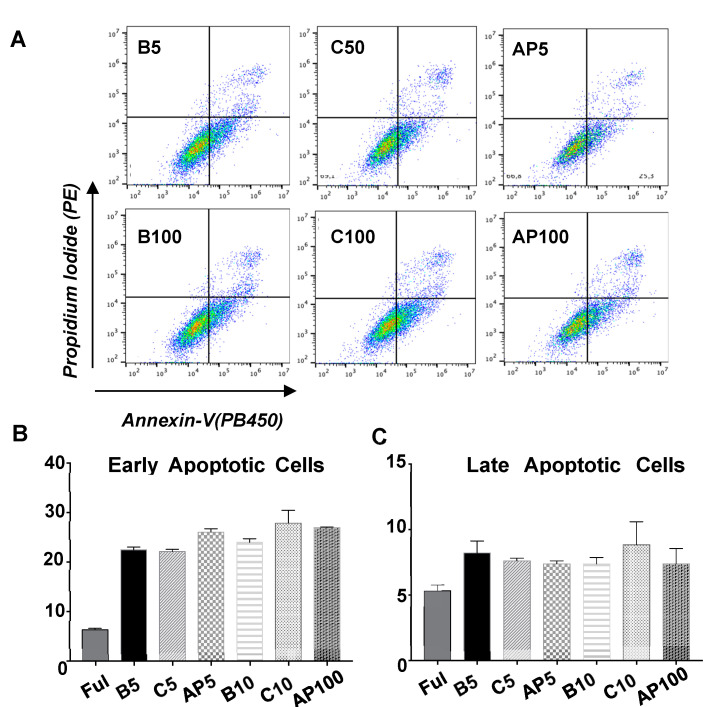
Effect of CAC secretome on ECFC apoptosis. ECFC were treated (n:3 per condition) with EBM-2 full (5% FBS + Growth factors, Full); serum free EBM-2 medium as basal condition (**B**), 50 or 100 ng/μL secretome of un-stimulated, control CAC (C50 and C100 respectively) and 50 or 100 ng/μL secretome of CAC pre-incubated whit AP factors (AP50 and AP100) for 24 h. Flow cytometry analysis was done using Annexin-V and Propidium Iodide. Graphs indicate the percentage of (**A**) representative dot-plots with propidium iodide (PE) and annexin-V (PB450 labelling) is shown for all conditions tested. (**B**) Early or (**C**) late apoptotic cells seen, represented as mean ± SE.

For all the conditions tested (Figure 5), ECFC showed higher apoptotic levels (especially early apoptosis) than ECFC incubated with EBM-2 complete medium (5% FBS and grow factors). Nevertheless, the incubation of ECFC with the secretomes of untreated CAC (C50, C100) or CAC after incubation with atherosclerotic factors (AP50, AP100), did not promote any significant increase of apoptosis (early or late) in these cells after 24 h, regardless secretome concentrations (50 or 100 ng/μL) compared to basal ECFC (B), indicating that at least, 24 h after their incubation with these factors, cell viability was not compromised.

## 3. Discussion

In the last years, there has been a significant increase in the number of pre-clinical and clinical studies focused on the application of EPC pursuing neovascularization in response to ischemia or CVD risk factors [12,14,15,28,29,30]. Moreover, EPC are also under consideration as a preventative and/or treatment strategy to provide vascular repair in atherosclerosis [4]. In response to vascular injury, EPC are recruited to the damaged area initiating an endogenous response to restore and replace the dysfunctional endothelium [4]. Unfortunately, despite their regenerative role, the number and function of EPC seem to be severely affected under pathological conditions, being essential to better understand how these cells work and how they react under such environments, in order to implement their use in regenerative cell therapies.

To date, two main sub-populations of the so-called EPC have been described: CAC, initially known as early EPC, and late EPC or ECFC. They both differ not only in their differentiation status, surface markers or their capability to form colonies; they also seem to contribute to neovascularization in different ways. Thus, while CAC are associated to a more paracrine role, by trophic support and enhancement of endogenous repair process [4,12], ECFC participate more directly by replacing damaged endothelial cells (EC) [10,31]. The “paracrine effect” of CAC has been assigned based on in vivo studies in which the cells themselves or their conditioned media administrated to murine models of critical limb ischemia or similar ones [12,13,32], induced an increase of vascular density or wound healing, without being able to detect these cells at the short term within the system [12]. Additionally, other studies have confirmed the presence of several pro-angiogenic factors in the conditioned media of EPC such as VEGF and GC-SF [33], PDGF-α, PDGF-β or KGF [32], CD163 and platelet factors such as CXCL4 and CXCL7 [34], among others. Moreover, full proteomic screenings of the EPC (both, CAC and ECFC) secretome have also been performed [34,35]. In the current study, we went a step further, evaluating, for the first time to our knowledge, the changes in the CAC secretome in response to an atherosclerotic environment. Thus, by using the same approach that we previously applied [5], healthy CAC were incubated for 2 h with the secretome of atherosclerotic arteries, identifying in total 92 up-regulated and 102 down-regulated proteins in the secretome of CAC in response to the atherosclerotic factors by a label-free proteomic approach. The changes detected correlated mainly, according to IPA, with CVD system development, cardiovascular diseases and inflammatory response, and more concretely with cell death and survival and also with an inhibition of angiogenesis and cell migration. Remarkably, the incubation of healthy ECFC with the conditioned media of CAC in presence/absence of atherosclerotic factors, corroborated, first, that CAC secretome promotes a significant increase of ECFC angiogenesis (mainly after 48 h), in agreement with previous studies [13], and secondly, that the secretome of “atherosclerotic” pre-conditioned CAC reverted such increase of angiogenesis, inhibiting it, independently of the time and dose tested, although these differences were slightly higher with 100 ng/µL CAC+AP secretome. Similarly, the secretome of un-stimulated CAC promoted a high increase of mobilization of ECFC, compared to ECFC basal, but such mobilization diminished in presence of pre-conditioned CAC. Our data were similar to previous results in which both angiogenesis and migration of ECFC decreased in response to CAC conditioned media in presence of antibodies against vascular endothelial growth factor (VEGF) and IL-8 [13]. In our study, the tendency was similar in all conditions tested (50 and 100 μg/mL at 24 h), but after 48 h ECFC mobilization was not that significant with higher levels of CAC factors. On the other hand, the incubation of ECFC with the secretome of CAC (untreated or under atherosclerotic conditions) did not promote any increase of cell apoptosis, at least after 24 h.

Angiogenesis, the formation of new vessels from pre-existing vasculature, is a complex and dynamic process that involves an orchestrated interplay between cells, ECM and soluble pro- and anti-angiogenic factors [36,37]. The balance between these factors determinates the increase, stabilization or reduction of the vascular network [38]. Angiogenesis participates in several physiological processes such as wound repair, but under healthy conditions the vasculature is remarkably quiescent. In a variety of diseases, atherosclerosis included, an angiogenic switch occurs tipping the balance in favor of de novo blood vessel formation [36]. Herein, our data clearly reflected an angiogenic switch, with the CAC secretome stimulating ECFC mediated angiogenesis, while under atherosclerotic conditions such process was blocked. Interestingly, an important set of proteins found as up-regulated in the “atherosclerotic” pre-conditioned CAC have been described as strong anti-angiogenic factors, which could explain the inhibitory effect. Many of them are ECM related proteins.

ECM plays a key role in the regulation of angiogenesis [39] and, moreover, in wound healing and tissue repair in response to injury [40]. ECM degradation, in response to diverse stimulus, leads to degradation or partial modification of matrix molecules, release of soluble peptides, “matrikines”, with pro- or anti-angiogenic activity [41]. Herein, several matrikines were up-regulated, probably by the action of proteases such as cathepsin D, matrix metallopeptidase 9 (MMP9), or similar molecules that are released in high levels by atherosclerotic plaques (as we and others have previously demonstrated [42]). Remarkably, many of the up-regulated proteins or peptides in CAC present anti-angiogenic properties, like the proteolytic fragments derived from the C-terminal cleavage of type XV and type XVIII collagens, restin and endostatin, respectively [43]. Both proteins inhibit bFGF-induced EC migration in vitro and exhibit anti-angiogenic properties in vivo [44]. Although they are both capable of suppressing tumor growth, endostatin has a stronger effect, being defined as one of the most potent endogenously produced angiogenic inhibitors [43]. Moreover, Endostatin inhibits VEGF-induced EC migration in a dose dependent manner [45]. Overall, endostatin and VEGF levels are considered the primary regulators of angiogenesis, whose levels may explain the balance between inhibition and stimulation of angiogenesis [43].

Similarly, heat shock protein beta-1 (HSPB1) [46], fibulins (FBLN1, FBLN2, FBLN5) [47,48,49,50], FN1 [51,52,53,54], or THBS1 [55] have also been shown to block angiogenesis by antagonizing or inhibiting VEGF angiogenic signaling pathways [46,47,49]. Also, THBS2, emilin 1, fibulins induce a blockade of angiogenesis, as well as cell migration and proliferation [49,56,57]. THBS1, for example, has been shown to be released in high concentrations by hypoxic EC, participating in blocking EC proliferation in vitro [58]. Different studies have described the interaction of many of these proteins between themselves, and also, for example, with other up-regulated proteins such as latent Transforming Growth Factor-beta (TGFβ) binding proteins (LTBP1, LTBP2, LTBP4) [59,60,61]. Remarkably, based on the differential proteins identified, IPA predicted an activation of TGFβ1 related pathways (67 identified proteins participate in TFGB1 signaling pathways, 41 of them indicated activation, Supplementary Table S4). Both, TGFβ and VEGF appear to be inter-connected in the regulation of angiogenesis, as well as main regulators of blood vessel development and maintenance [61,62,63,64], and the interaction between themselves and other proteins identified here has been largely described [49,57,62,65]. Further assays should be carried out in order to confirm the involvement of such pathways in the angiogenic switch induced by the AP secretome over the CAC secretion profile.

Apart from all these ECM related proteins, the anti-angiogenic role of other up-regulated proteins such as C3 [66], SERPINF1 [67] or HTRA1 [68], has also been described in both in vitro and in vivo assays. Finally, several Apo-lipoproteins (APOA1, APOH, APOE) were also highly released by CAC in response to the atherosclerotic plaque secretome. APOA1 has been shown to inhibit angiopoietin-1 and VEGF-mediated tube formation in HUVECs, by modulating the plasminogen system [69]. In addition, APOE, apart from mediating the clearance of different lipoproteins from circulation, has also been shown to inhibit in vitro HUVEC cell migration and tube formation at low concentrations, by a peptide called apo-Edp, derived from the APOE residues 141–149 [70,71].

On the other hand, proteins known for their angiogenic potential, such as HMOX1 or HSP90, were down-regulated in the secretome of CAC+AP. Indeed, different HSP90 inhibitors are currently under study as anti-angiogenic strategy in tumor growth [72]. Moreover, down-regulation of HMOX1 has been correlated with a decreased proliferation, migration, formation of capillaries, and paracrine proangiogenic potential of bone marrow-derived cells [73].

Overall, proteomic and functional results corroborated the data previously published [5], indicating that the atherosclerotic factors induce in CAC changes that might resemble the initial response to the atherosclerotic damage. Our data indicated that the secretome of pre-stimulated CAC with the atherosclerotic factors decreased migration and tubule formation of ECFC. Initially, this could represent an impairment of CAC pro-angiogenic role and therefore an impairment of ECFC regenerative properties. In terms of atherosclerosis, however, such inactivation might be beneficial taking into account that angiogenesis appears to be a key process in the development of atherosclerosis [74]. Indeed, angiogenesis supports initial plaque growth increasing the support of oxygen and nutrients to the artery wall, contributing to plaque growth and stabilization in early lesions [38,75]. Once the atherosclerotic plaque develops, continued angiogenesis results in an increased density of micro-vessels and structurally and functionally abnormal vessels [76], promoting vessel leakage, plaque destabilization, and thromboembolic events at the later stages [38,77]. In this regard, a few in vivo studies have shown the potential of using angiogenic inhibitors in order to block vessel formation and slow down the progression of atherosclerotic lesion formation and reduce plaque size [38,75,78]. For example, the use of endostatin was able to reduce neo-vessel formation and concomitant aortic plaque growth in APOE deficient mice [75,79]. These results suggest that anti-angiogenic therapy may have beneficial effects in patients by modulating plaque vascularization. On the other hand, an excessive presence of anti-angiogenic agents may destroy abnormal vessels but also affect the vasculature of some normal tissues [76]. Thus, while several studies support the use of anti-angiogenic therapy in atherosclerosis [76,78,80], an appropriate dose of such anti-angiogenic factors should be found, in order to achieve a “normalized” vasculature, less leaky vessels but stabilizing immature vessels [76,81,82]. In addition, the studies with angiogenic inhibitors have been mainly focused on a single target, e.g., anti-VEGF or anti-Angiopoietin-2 monotherapies [80,83], but it is not clear whether monotherapy will be sufficient for prolonged normalization of intra-plaque vessels or, on the contrary, if various angiogenic factors should be targeted [63,76].

Herein, CAC pre-conditioning with atherosclerotic factors affected their pro-angiogenic role, promoting the release by these cells of an important number of anti-angiogenic factors, which in turn, reduced the capacity of ECFC to form tubules in a Matrigel-based approach, without compromising, at least in the early stages, cell viability. Perhaps, in the same way that the conditioned medium of CAC has been proposed to be administrated instead of cells themselves in ischemic revascularization approaches, the use of the pre-conditioned media of CAC containing such anti-angiogenic cocktail, as described here, might be an alternative to modulate excessive angiogenesis in atherosclerosis. Future research will allow for confirming such a hypothesis.

## 4. Materials and Methods

### 4.1. Sample Acquisition

Circulating angiogenic cells, hereafter referred to as CAC, were isolated from buffy coats from healthy donors provided by the Andalusian Biobank Network (Decree 1/2013). Carotid atherosclerotic segments were obtained from patients undergoing endarterectomy at the University Hospital Puerta del Mar, Cadiz. All volunteers provided informed consent prior to sample collection. This study was approved by the local Ethics Committee, in accordance to Spanish and European Union Regulations and it follows the principles outlined in the Declaration of Helsinki.

### 4.2. CAC Isolation and Culture

CAC were isolated from peripheral blood mononuclear cells and cultured as previously described [5,12]. Briefly, 10^6^ mononuclear cells were plated in fibronectin coated plates and incubated in EBM-2 media plus 5% fetal bovine serum (FBS) and SingleQuots growth factors (Lonza, Basel, Switzerland). Non-adherent cells were discarded after four days and attached cells were allowed to grow in fresh media until day 7, when experimental assays were performed.

### 4.3. Atheroma Plaque Isolation and Culture

Carotid atherosclerotic arteries (n:7) were cultured as described [5,84]. Briefly, the atherosclerotic/non-thrombosed regions were selected and washed several times with PBS 1X first and later on with RPMI 1640 medium containing 1% penicillin/streptomycin (P/S) for 24 h, and further incubated with RPMI medium 72 h, at 5% CO_2_, 37 °C. Supernatants were then collected and centrifuged to remove tissue debris, protein concentration was measured with BCA (Pierce^TM^ BCA Protein Assay Kit), and supernatants were immediately used or snap-frozen in liquid nitrogen prior to storage at −80 °C.

### 4.4. Characterization of CAC

A flow cytometry assay was carried out to confirm CAC identity, using specific antibodies against CD31, CD34, CD45, CD105, CD90, CD73, CD309, CD105, CD133, CD146, and CD14 molecular markers, as described [12]. An isotype IgG1 antibody was used for negative control. A Cyto-FLEX instrument (Beckman Coulter, Brea, CA, USA) was used for flow cytometry and data was processed with CytExpert 1.0 software (Beckman Coulter). The full list of antibodies is shown in Supplementary Table S5, and a Supplementary Figure S1 includes CAC characterization.

### 4.5. CAC Incubation Ex Vivo with Atheroma Plaque Secretome

At day 7 after CAC isolation, the conditioned media was removed and cells were incubated overnight, 5% CO_2_ at 37 °C, without FBS, to avoid serum proteins in the supernatant. After that, cells were washed with PBS 1× and incubated for 2 h (37 °C, 5% CO_2_) with either a mixture of 50 μg of proteins from the atheroma plaque (AP) secretome and basal medium without FBS (CAC+AP, or AP) or only with basal medium (CAC Control, or C). Subsequently, conditioned media was discarded and both sets of CAC were incubated again for 5 h with EBM-2 medium without FBS. Supernatants from both, CAC+AP and CAC controls, were collected and centrifuged at 1500× *g* for 5 min, 4 °C, to remove cell debris, and then the secretome was concentrated by centrifugation with AMICON15 (Millipore), 4000× *g* for 1 h (4 °C). Finally, protein concentration was quantified with BCA, following the kit protocol (Pierce^TM^ BCA Protein Assay Kit).

### 4.6. Proteomic Analysis

In total, 100 μg of proteins per sample (CAC control and CAC+AP secretomes, n:3 per condition) were precipitated with 100% acetone, overnight, at −20 °C. Pellets were collected by centrifugation at 13,300 rpm for 20 min, 4 °C, and resuspended in 6 M urea for further tryptic in-solution digestion. Proteins were reduced (200 mM Dithiothreitol, 45 min) and alkylated (1M Iodoacetamide, 45 min) before being diluted four times with 50 mM ammonium bicarbonate and digested with trypsin (enzyme/substrate ratio 1:50) at 37 °C over-night. The reaction was quenched with 0.1% TFA and resulting peptides were desalted using C18 stage-tips.

Peptide samples were analyzed (2 technical replicates) by liquid chromatography coupled to tandem mass spectrometry (LC-MS/MS), using a nano LC-Ultra 1D+ system (Eksigent, Dublin, CA, USA) coupled to an Impact mass spectrometer (Bruker, Billerica, MA, USA) via a captive-spray source (Bruker) supplemented with a nano-Booster operated at 0.2 bar/min with isopropanol as dopant. Peptides were loaded into a trap column (NS-MP-10 BioSphere C18, 5 µm, 20 mm length, Nanoseparations) for 10 min at a flow rate of 2.5 µL/min in 0.1% formic acid (FA). Then peptides were transferred to an analytical column (ReproSil Pur C18-AQ 1.9 µm, 400 mm length and 0.075 mm ID) and separated using a 95 min effective linear gradient (buffer A: 4% acetonitrile (ACN), 0.1% FA; buffer B: 100% ACN, 0.1% FA) at a flow rate of 250 nL/min. The gradient used was: 0–2 min 4% B, 2–80 min 30%B, 80.5–87.5 min 98% B, 88–95 min 2% B. Peptides were electrosprayed (1.35 kV) into the mass spectrometer with a heated capillary temperature of 180 °C. The mass spectrometer was operated in a data-dependent mode, with an automatic switch between MS (80–1600 *m/z*) and MS/MS (80–1600 *m/z*) scans using a top 30 method (threshold signal ≥ 500 counts, z ≥ 2 and *m/z* ≥ 350). An active exclusion of 30 s was used. Precursor intensities were re-evaluated in the MS scan (n) regarding their values in the previous MS scan (n-1). Any *m/z* intensity exceeding 5 times the measured value in the preceding MS scan was reconsidered for MS/MS. Peptides were isolated using a 2 Th window and fragmented using collision induced dissociation (CID) with a collision energy of 23–56 eV as function of the *m/z* value. In addition, to ensure an accurate reproducible label-free quantification data across the entire set of samples, a quality control (QC) sample (human HEK293 cell line digest) was run at the beginning and at the end of the experiment, and also every 5 samples. No significant variations in the performance of the LC-MS/MS instrument were observed.

Raw files were processed with MaxQuant (v 1.6.0.1) using standard settings searching against a human protein database (UniProtKB/Swiss-Prot) supplemented with contaminants. Carbamido-methylation of cysteines, oxidation of methionine, protein N-term acetylation were set as variable modifications. Minimal peptide length was set to 7 amino acids and a maximum of two tryptic missed-cleavages were allowed. Results were filtered at 0.01 FDR (peptide and protein level). Label-free quantification was done with match between runs (match window of 0.7 min and alignment window of 20 min). Afterwards, the “proteinGroup.txt” file was loaded in Perseus (v1.6.0.2) for further statistical analysis. A minimum of four LFQ valid values per group was required for quantification. Missing values were imputed from the observed normal distribution of intensities. Then, a two-sample student’s *T*-test with a permutation-based FDR was performed. For AP group vs. control group, only proteins with a *q*-value < 0.05 and log_2_ ratio >1.35 or <−1.35 were considered as differentially up- or down-regulated.

Finally, Ingenuity Pathway Analysis (IPA) software was applied to evaluate the functional roles of the identified proteins.

### 4.7. Validation of Protein Expression Changes

The levels of thrombospondin-1 (THBS1) and apolipoprotein-E (APOE) were measured by Western blot following standard procedures. Proteins (30 μg) from different supernatants were diluted into Laemli buffer (50 mM Tris pH 6.8, 10% *v/v* glycerol, 2% *w/v* SDS, 0.1% *w/v* bromophenol blue), heated for 5 min at 95 °C, and separated on 4–15% Mini-PROTEAN TGX Stain-Free Gel (BioRad, Hercules, CA, USA), whose image was taken for loading control, and transferred onto a PVDF membrane employing the Trans-Blot^®^ Turbo^TM^ Transfer System (BioRad). The membrane was blocked for 1 h with 5% bovine serum albumin and immunoblotted with the primary rabbit-anti-THBS1 (1:500) and goat-anti-APOE (1:3000) antibodies over-night, at 4 °C, followed by incubation with the respective secondary antibodies, for 1 h. Both, stain free gels and blots were imaged employing a ChemiDoc^TM^ Touch Imaging System (BioRad). Detailed information regarding antibodies can be found in Supplementary Table S5.

### 4.8. ECFC Isolation and Culture

ECFC were isolated from umbilical cord blood (Cb-ECFC) and cultured as previously described [85,86]. Briefly, mononuclear cells were plated in on fibronectin-coated 6-well tissue culture plates (BD Bioscience, San Jose, CA, USA) using endothelial cell-medium (EBM-2 without hydrocortisone, Lonza; 20% FBS; 1X glutamine-P/S). Non-adherent cells were discarded after 72 h and the bound fraction maintained in endothelial cell-medium, with media being replenished every 2–3 days. Attached cells were allowed to grow in fresh media until day 27. Endothelial colonies were identified as well-circumscribed monolayers of ≥60 cells with cobblestone morphology. Cb-ECFC were cryopreserved in aliquots equivalent to 10^6^ cells/mL-freezing medium (Invitrogen, Carlsbad, CA, USA, 12648-010) and stored in liquid nitrogen or directly cultured on 1% gelatin-coated plates using ECFC-medium: EBM-2 (except for hydrocortisone; Lonza) supplemented with 5% FBS, 1X P/S, and SingleQuots growth factors (Lonza) for further assays. ECFC between passages 5 and 7 were used for all experiments.

### 4.9. Characterization of ECFC

ECFC phenotype was characterized by testing cloning-forming ability, migration towards fibroblast growth factor-2 (FGF-2), angiogenesis assays and indirect immunofluorescence. Details can be found in expanded Materials and Methods in the online-only Data Supplement, as well as a figure including all characterization steps performed (Supplementary Materials and Methods and Supplementary Figure S2).

### 4.10. Angiogenesis Assay

A Matrigel-tube formation assay was carried out to assess the effect of CAC secretomes (previously stimulated or not with atherosclerotic factors) over ECFC angiogenic potential. Briefly, ECFC (15 × 10^3^) were seeded into a µ-Plate Angiogenesis 96 Well (ibidi, 89646) pre-coated with 10 μL of Matrigel™ (BD Bioscience, 356231), and incubated, in triplicates, with 50 µL (total volume) of: 50 and 100 ng/µL of the secretome of control CAC, in EBM-2 basal medium (C50 and C100, respectively, n:3); the secretome of CAC pre-stimulated with atherosclerotic factors, 50 and 100 ng/µL in basal medium (AP50 and AP100, n:3); or EBM-2 basal medium with equivalent amounts of SingleQuots growth factors (Lonza), considered as control, un-treated ECFC (B50 and B100, n:3). In addition, a positive control with 15 × 10^3^ ECFC plus 35 mg/mL of the angiogenic activator recombinant human fibroblast growth factor (FGF, n:2) (#233-FB, R& D Systems), and a negative control with ECFC plus the inhibitor 5 µM sulforaphane (SR, n:2) (Merck, Kenilworth, NJ, USA, #S4441) were also included in the assay. ECFC were then cultured for 48 h, at 37 °C, 5% CO_2_. Images of tubule formation were collected per well at 0, 24, and 48 h using a Moticam 3.0 camera connected to an inverted phase-contrast microscope, under 40× magnification. The total number and area of meshes per well, total number of segments, and the total length of the segments were quantitated from captured images using the Angiogenesis Analyzer plugin using Image J v2.0.

### 4.11. Wound Migration Assay

Culture-insert 2 wells (#81176, ibidi) were used to study ECFC migration in response to CAC secretomes. Following manufacturer’s instructions, 2 × 10^4^ ECFC in EBM-2 basal medium were seeded into each well of the culture-insert, pre-coated with gelatin 1%, and cultured at 37 °C, 5% CO_2_, allowing ECFC to get attached and confluent. After 24 h, culture-inserts were detached, the cell medium discarded and cells were then incubated 48 h with serum free EBM-2 basal medium with equivalent amounts of SingleQuots growth factors (Lonza) (Basal ECFC, B50 and B100, n:3); with 50 and 100 ng/µL of the secretome of control CAC (C50 and C100, respectively, n:3) in EBM-2 basal medium; or with 50 and 100 ng/µL of the secretome of CAC pre-stimulated with atherosclerotic factors (AP50 and AP100, n:3). Images were taken at baseline (time 0), and after 24 and 48 h under a phase contrast microscope, as indicated before. Cell migration was measured using NIH ImageJ v2.0, calculating the percentage area of wound closure after 24 and 48 h versus baseline.

### 4.12. Apoptosis Assay

The paracrine effect of CAC over ECFC apoptosis was evaluated by flow cytometry. Briefly, ECFC (7 × 10^4^ cells/well) were seeded in 24-well plates and allowed to settle 24 h, at 37 °C, 5% CO_2_. Cell medium was then discarded and ECFC were incubated another 24 h with either EBM-2 basal medium, without FBS and equivalent amounts of SingleQuots growth factors (Lonza) (Basal ECFC, B50 and B100, n:3); with 50 and 100 ng/µL of the secretome of control CAC (C50 and C100, respectively, n:3) in EBM-2 basal medium; or with 50 and 100 ng/µL of the secretome of CAC pre-stimulated with atherosclerotic factors (AP50 and AP100, n:3). ECFC were detached with Accutase 1× and incubated with annexin-V (PB-V450, #560506, BD) and propidium iodide (IP, #556463, BD) at 4 °C, 30 min. Fluorescence was measured with a Cytoflex cytometer (Beckman Coulter) and the CytoExpert software. Finally, data were analyzed with FlowJo v 10.4 software.

### 4.13. Statistical Analysis

Protein-related statistics were obtained with MaxQuant and IPA software, while functional-related statistical analysis was performed with GraphPad Prism 7 software. The *p*-value provided by IPA was calculated using the right-tailed Fisher Exact Test, and it represents the likelihood that the association between a set of focus genes in your experiment and a given process or pathway is due to random chance. For functional assays, data were verified for normal distribution using Shapiro–Wilk normality test. Differences between groups were assessed with two-way ANOVA test completed with Tukey-s multiple comparisons test for post hoc analyses. Data were represented as mean ± SE and differences were considered statistically significant at *p*-value < 0.05.

## 5. Conclusions

**Figure 6:**
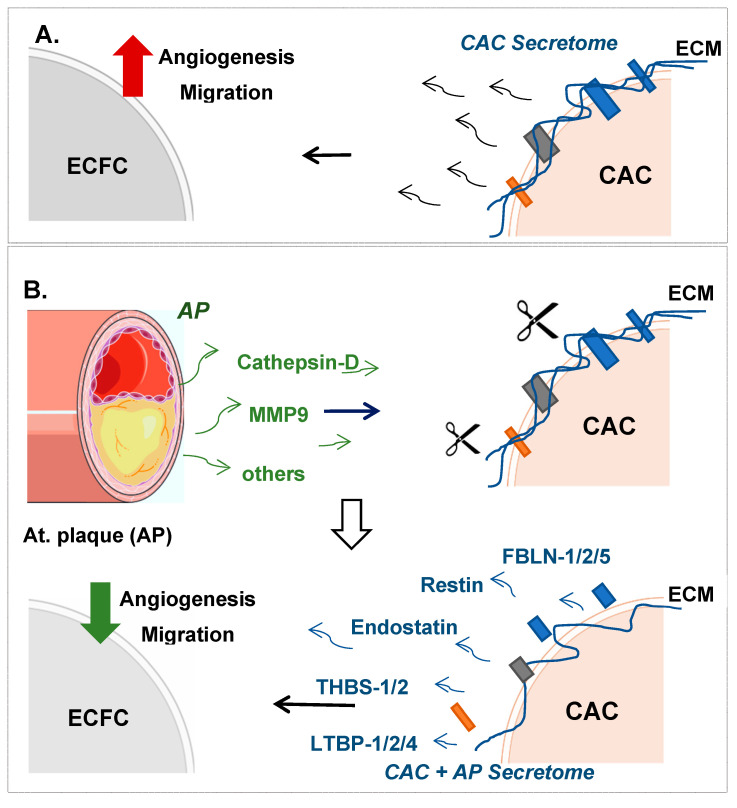
CAC paracrine effect over ECFC in response to atherosclerotic factors. The current figure shows a schematic overview of the processes taking place in response to the direct contact of CAC with the atherosclerotic plaque secretome (AP). (**A**) The secretome of CAC induce (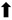) ECFC angiogenesis and migration. (**B**) Incubation of CAC with atherosclerotic factors (Cathepsin-D, MMP9 and other proteases) promotes the rupture and degradation of ECM proteins. Further incubation of ECFC with the CAC released factors (fibulins, endostatin, restin, thrombospondins, LTBP1, etc.) inhibits the paracrine effect of CAC, since angiogenesis and migration of ECFC decreases (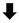) compared to ECFC stimulated with the secretome of control CAC (not affected by AP factors). Curved arrows represent the release of factors by CAC, the AP arteries or CAC after incubation with AP secretome. Straight arrows indicate the direction of the effect of the factors released by CAC control (**A**), AP or CAC+AP (**B**).

We have shown here, for the first time, the effect of atherosclerotic factors over the paracrine role of CAC ex vivo (Figure 6). Our data indicated that the action of proteins secreted in high levels by atherosclerotic plaques such as proteases like Cathepsin D or MMP9 [42], promotes the release by CAC of several proteins or peptide fragments mainly from the ECM and basement membrane (fibulins, restin, endostatin, thrombospondins, latent TGFβ binding proteins, among others). Many of these proteins have been described as strong anti-angiogenic factors, and they might be responsible for promoting an angiogenic switch by impairing ECFC tubule formation ex vivo, as we saw in our functional assays. Future research should be performed to confirm whether the secreted anti-angiogenic factors of pre-conditioned CAC under atherosclerotic conditions also promote a blockade of ECFC neovascularization in vivo, impairing its regenerative role or, as many authors suggest, they could contribute to modulating and reducing the atherosclerotic process.

## Figures and Tables

**Table 1:** Functional and disease classification of proteins released only in CAC secretome in response to atherosclerotic factors. Protein classification was made with Ingenuity Pathway Analysis (IPA) software. The table includes the main categories containing related functions or diseases, predicted activation (↑) or inhibition (↓) according to IPA, number of molecules per category, *p*-values assigned, and protein names. Legend: up-regulated ↑, down-regulated ↓. EC: Endothelial cell; CV: Cardiovascular.

	Diseases or Functions	Molecules	*p*-Value	Gene Names
**CV System Development**	**EC Movement** 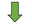	20	1.01 × 10^−8^	↑APOE, ↑APOH, ↑CDH13, ↑COL18A1, ↑FN1, ↑HSPB1, ↑HSPG2, ↑KNG1, ↑ORM1, ↑SERPINF1, ↑THBS1, ↑THBS2, ↑VTN, ↓G6PD, ↓GPI, ↓HSP90AB1, ↓ITGB2, ↓MARCKS ↓PTPN6, ↓TMSB10
	**EC Development** 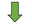	16	4.54 × 10^−6^	↑APOA1, ↑APOE, ↑APOH, ↑C3, ↑CDH13, ↑COL18A1, ↑F2, ↑FN1, ↑HSPG2, ↑IGHG1, ↑KNG1, ↑SERPINF1, ↑THBS1, ↑VTN, ↓G6PD, ↓HMOX1
	**EC Migration** 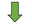	18	7.64 × 10^−8^	↑APOE, ↑APOH, ↑CDH13, ↑COL18A1, ↑FN1, ↑HSPB1, ↑KNG1, ↑ORM1, ↑SERPINF1, ↑THBS1, ↑VTN, ↓G6PD, ↓GPI, ↓HSP90AB1, ↓ITGB2, ↓MARCKS ↓PTPN6, ↓TMSB10
	**Vasculogenesis** 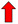	37	4.10 × 10^−13^	↑APOA1, ↑APOE, ↑APOH, ↑C3, ↑C6, ↑CDH13, ↑CFB, ↑COL15A1, ↑COL18A1, ↑F2, ↑FBLN1, ↑FBLN5, ↑FN1, ↑HSPG2, ↑HTRA1, ↑IGFBP7, IGHG1, ↑KNG1, ↑LTBP1, ↑MYH10, ↑ORM1, ↑SERPINF1, ↑TGM2, ↑THBS1, ↑THBS2, ↑TNC, ↑VTN, ↓CAPN1, ↓CYCS, ↓GCLC, ↓GLRX, ↓G6PD, ↓HMOX1, ↓ITGB2, ↓PTPN6, ↓STX7, ↓YARS
	**Angiogenesis** 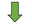	41	6.80 × 10^−13^	↑APOA1, ↑APOE, ↑APOH, ↑C3, ↑C6, ↑CDH13, ↑CFB, ↑COL15A1, ↑COL18A1, ↑EMILIN1, ↑F2, ↑FBLN1, ↑FBLN2, ↑FBLN5, ↑FN1, ↑HSPB1, ↑HSPG2, ↑HTRA1, ↑IGFBP7, ↑IGHG1, ↑KNG1, ↑LTBP1, ↑MYH10, ↑ORM1, ↑SERPINF1, ↑TGM2, ↑THBS1, ↑THBS2, ↑TNC, ↑VTN, ↓CAPN1, ↓CYCS, ↓GCLC, ↓GLRX, ↓G6PD, ↓HMOX1, ↓HSP90AA1, ↓ITGB2, ↓PTPN6, ↓STX7, ↓YARS
**Cell Death and survival**	**Necrosis** 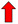	92	2.54 × 10^−23^	↑APOE, ↑APOA1, ↑BGN, ↑C3, ↑C7, ↑C9, ↑CD59, ↑CD5L, ↑CFH, ↑CLU, ↑COL6A1, ↑COL18A1, ↑EFEMP1, ↑F2, ↑FBLN1, ↑FMOD, ↑FN1, ↑HIST1H2BO, ↑HSPB1, ↑HTRA1, ↑IGFBP7, ↑IGHG1, ↑IGHM, ↑KNG1, ↑LMNA, ↑LTBP1, ↑LUM, ↑MYH10, ↑MYH11, ↑PDLIM7, ↑POSTN, ↑SERPINF1, ↑SOD3, ↑SRI, ↑THBS1, ↑TNC, ↑THBS2, ↑TGM2, ↑TPM1, ↑TTR, ↑VCAN, ↑VTN, ↓ALDH1A1, ↓ADH5, ↓AP2A2, ↓AP2M1, ↓ARCN1, ↓BCAP31, ↓CAPN1, ↓CAST, ↓CCT5, ↓CCT6A, ↓CLTC, ↓CSTB, ↓CYCS, ↓DYNC1H1, ↓G6PD, ↓GCLC, ↓GLRX, ↓GNAI2, ↓GPI, ↓HMOX1, ↓HSP90AA1, ↓HSP90AB1, ↓IDH2, ↓ITGB2, ↓LGMN, ↓LYN, ↓MVP, ↓NAMPT, ↓PSMB1, ↓PSMC1, ↓PNP, ↓PSMC6, ↓PTPN6, ↓PTPRC, ↓PYCARD, ↓RACK 1, ↓RPL10, ↓RPL9, ↓RPL34, ↓RPS13, ↓SERPINB2, ↓STIP1, ↓SNX1, ↓S100A11, ↓TCP1, ↓TTN, ↓TMSB10, ↓TUFM, ↓UBE2L3, ↓YARS
	**Apoptosis** 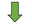	84	2.10 × 10^−18^	↑APOA1, ↑APOE, ↑BGN, ↑C3, ↑C6, ↑CD59, ↑CD5L, ↑CLU, ↑COL18A1, ↑EFEMP1, ↑F2, ↑FBLN1, ↑FBN1, ↑FMOD, ↑FN1, ↑HTRA1, ↑HSPB1, ↑HSPG2, ↑IGFBP7, ↑IGHG1, ↑IGHM, ↑KNG1, ↑LMNA, ↑LTBP1, ↑LUM, ↑MYH10, ↑MYH11, ↑PDLIM7, ↑VCAN, ↑TTR, ↑SRI, ↑CFH, ↑TPM1, ↑SERPINF1, ↑SOD3, ↑TGM2, ↑THBS1, ↑THBS2, ↑TNC, ↑VCL, ↑VTN, ↓ADH5, ↓ALDH1A1, ↓AP2A2, ↓AP2M1, ↓ATOX1, ↓BASP1, ↓BCAP31, ↓CANX, ↓CAPN1, ↓CAST, ↓CLTC, ↓CSTB, ↓CYCS, ↓DYNC1H1, ↓GCLC, ↓GLRX, ↓GNAI2, ↓G6PD, ↓GPI, ↓HMOX1, ↓HSP90AA1, ↓HSP90AB1, ↓ITGB2, ↓LGMN, ↓LYN, ↓MVP, ↓NAMPT, ↓PSMB1, ↓PTPN6, ↓PTPRC, ↓PYCARD, ↓PNP, ↓RACK1, ↓RPL10, ↓RRBP1, ↓S100A11, ↓SERPINB1, ↓SNX1, ↓STIP1, ↓TCP1, ↓TMSB10, ↓TTN, ↓YARS
**CV Disease**	**Atherosclerotic Lesion** 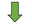	11	1.13 × 10^−6^	↑APOA1, ↑APOE, ↑CD59, ↑COL18A1, ↑FN1, ↑HSPG2, ↑IGHG1, ↑LPA, ↑THBS1, ↑VCAN, ↓HMOX1
	**Vascular Lesion** 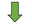	16	7.59 × 10^−9^	↑APOA1, ↑APOE, ↑CD59, ↑COL18A1, ↑FBN1, ↑FN1, ↑HSPG2, ↑IGFBP7, ↑IGHG1, ↑LPA, ↑MYH11, ↑THBS1, ↑VCAN, ↓HMOX1, ↓PTPRC, ↓RRBP1
**Inflammatory response** 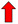		38	9.27 × 10^−14^	↑APOA1, ↑APOE, ↑APOH, ↑C3, ↑C6, ↑CFH, ↑COL18A1, ↑F2, ↑FN1, ↑HSPB1, ↑HSPG2, ↑IGHG1, ↑IGHM, ↑KNG1, ↑LPA, ↑LTBP1, ↑LUM, ↑ORM1, ↑PGLYRP2, ↑SERPINF1, ↑SOD3, ↑TGM2, ↑THBS1, ↑THBS2, ↑TNC, ↑VTN, ↓GNAI2, ↓HEBP1, ↓HMOX1, ↓ITGB2, ↓LGMN, ↓LYN, ↓PPIB, ↓PTPN6, ↓PYCARD, ↓SERPINB1, ↓TMSB10, ↓YARS

## Supplementary Materials

Supplementary Materials can be found at https://www.mdpi.com/1422-0067/21/15/5256/s1. MS data have been deposited into the ProteomeXchange Consortium via the Pride [87] partner repository with the dataset identifier PXD019395.

## Abbreviations

AP	Atheroma plaque
APO	Apo-lipoproteins
APOE	Apolipoprotein-E
B	Basal
C	Control
CAC	Circulating angiogenic cells
Cb-ECFC	umbilical cord blood endothelial colony forming cell
CID	Collision induced dissociation
COL15A1	Restin
COL18A1	Endostatin
CVD	Cardiovascular diseases
EBM-2	Endothelial Basal Medium-2
EC	Endothelial cells
ECFC	Endothelial colony-forming cells
ECM	Extracellular matrix
EPC	Endothelial progenitor cells
eEPC	early EPC
FA	Formic acid
FBLN	Fibulin
FDR	False Discovery Rate
FGF	Fibroblast Growth Factor
FN1	Fibronectin-1
GC-SF	Granulocyte colony-stimulating factor
HMOX1	Heme Oxygenase-1
HTRA1	HtrA Serine Peptidase-1
HSP90	Heat shock protein 90
HSPG2	Heparan Sulfate Proteoglycan-2
HUVEC	Human umbilical vascular endothelial cell
IPA	Ingenuity Pathway Analysis
KGF	Keratinocyte growth factor
LC-MS/MS	Liquid Chromatography- Tandem Mass spectrometry
LFQ	Label Free Quantitation
LTBP	Latent TGF-beta binding proteins
MAC	Myeloid angiogenic cells
MMP9	Matrix metallopeptidase-9
MS	Mass spectrometry
PBS	Phosphate buffer saline
PCA	Principal Component Analysis
PDGF	Platelet-derived growth factor
P/S	Penicillin/streptomycin
SR	Sulforaphane
THBS	Thrombospondin
THBS1	Thrombospondin-1
TFA	Trifluoroacetic acid
TGFβ	Transforming Growth Factor-beta
TNC	Tenascin
VEGF	Vascular Endothelial growth factor
