# Supplementary material for: Atherosclerotic Pre-Conditioning Affects the Paracrine Role of Circulating Angiogenic Cells Ex-Vivo
Source: Int J Mol Sci. 2020 Jul 24;21(15):5256. doi: 10.3390/ijms21155256 (PMC7432497; doi:10.3390/ijms21155256)
Supplement: Supplementary file 1 [file ijms-21-05256-s001.pdf]

Supplementary Materials and Methods

"Atherosclerotic pre-conditioning affects the paracrine role of circulating angiogenic cells *ex-vivo*"

Characterization of ECFC

ECFC phenotype was characterized by testing cloning-forming ability, migration towards FGF-2, angiogenesis assays and indirect immunofluorescence.

1. Clonal ability

ECFC were cultured for 10 days at clonal density (10 cell/cm^2) onto 1% gelatin-coated plates using endothelial cell-medium. Medium was refreshed on day 5. At day 10, colonies containing 12 or more cells (i.e., at least 3 divisions) were scored under a fluorescence microscope after Hoechst (1:1000; Life technologies, video time lapse, Leica) staining of the nuclei. Endothelial phenotype was confirmed through the binding of FITC labeled Ulex Europaeus Agglutinin I (UEA-1; 2:100; Vector Laboratories, Burlingame) using a fluorescence microscope. Colonies were categorized into large (>300 cells), medium (101- 300 cells) and small (12-100 cells) sizes. The number of cells in each colony was quantified under a fluorescence microscope after Hoechst staining using ImageJ analysis software.

2. Scratch assay

Confluent cultures of ECFC were pre-incubated in EBM-2, 5 % FBS (basal medium) for 6 h. Scratch wounds were generated across using a pipette tip. Cells were then treated for 24 h using basal medium in the presence or absence of 1 ng/mL FGF-2. Scratch size was measured after 24 h for each culture condition with phase contrast microscope

3. Indirect immunofluorescence

Immunofluorescence was carried out using mouse anti-human CD31 (1:100; BD Biosciences, REF: 130-092-653) and mouse anti-human von Willebrand factor (vWF) (1:200; Genetex), followed by Alexa Flour 488 donkey anti-mouse conjugated secondary antibody (1:1000; Life technologies, REF:A-21202) and cell nuclei were counterstained with Hoechst. Also, endothelial phenotype was confirmed through the

binding of FITC labeled Ulex Europaeus Agglutinin I (UEA-1; 2:100; Vector Laboratories, Burlingame) using a fluorescence microscope.

4. Angiogenesis assays

The formation of vascular networks in vitro was evaluated using Matrigel. ECFC were seeded on Matrigel-coated plates at a density of $2x10^4$ cell/cm^2 and incubated for 24h in endothelial cell-medium. The lengths of ECFC-lined cords were visualized under a fluorescence microscope.

Supplementary figure S1

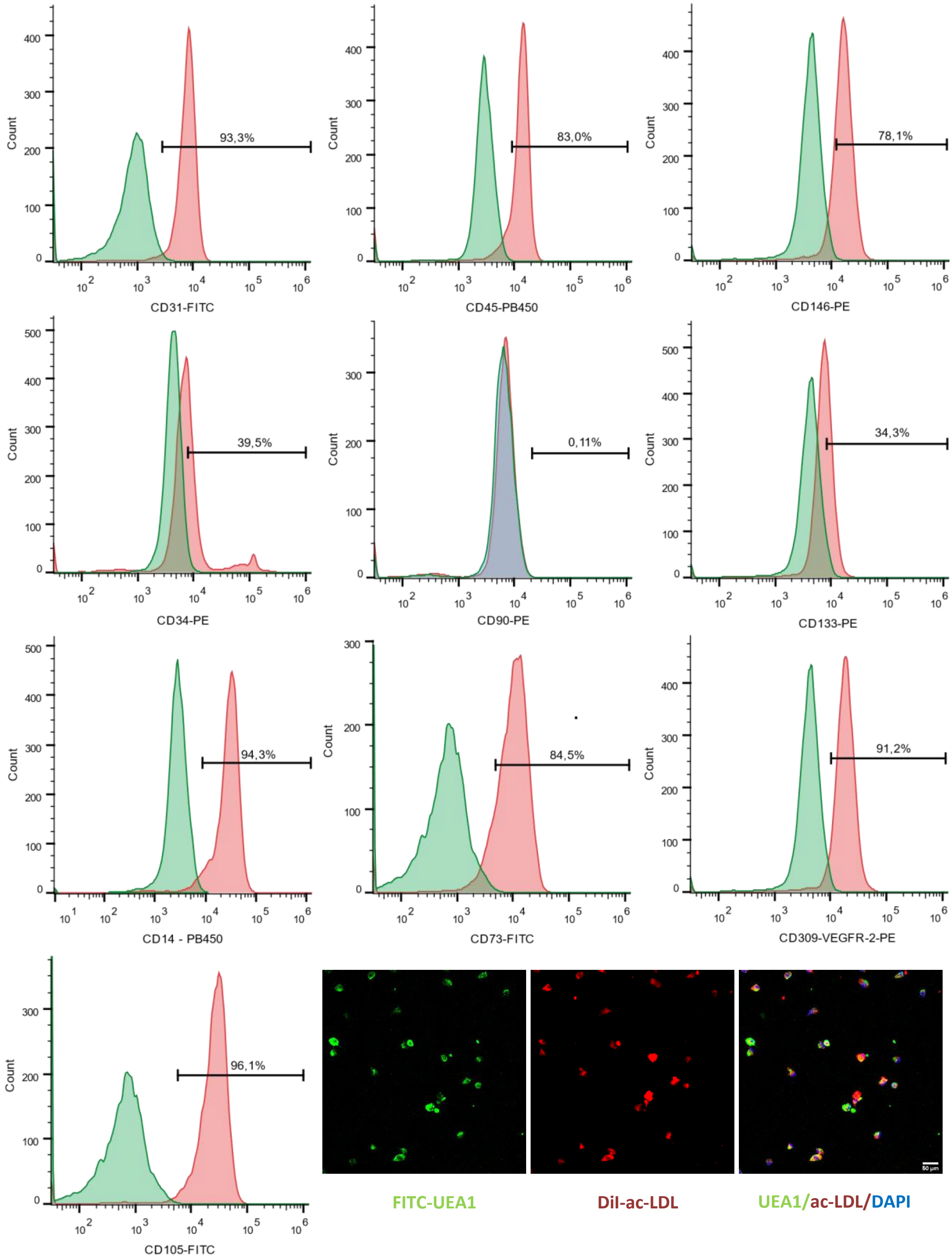

Supplementary Figure S1: Characterization of cultured CAC. Cells were isolated from PBMC of healthy donors and cultured in fibronectin-coated plates. On day 7, cell identity was confirmed by flow cytometry, analyzing the following markers: CD31, CD34, CD45, CD90, CD73, CD105, CD309 (VEGFR2), CD133, CD146 and CD14. Specific isotype antibodies were used as negative controls. Data were presented as percentage of positive cells (%), for both, the isotype control (blue) and positive markers analyzed (red). Additionally, CAC identity was also confirmed by immunohistochemistry, with cells presenting double labeling with UEA1 and Ac-LDL uptake.

Supplementary Figure S2: Characterization of ECFC. (A) Cloning-forming ability of ECFC was examined at clonal density (passage 1). The endothelial nature of the colonies was confirmed by binding of UEA-1 lectin (green) and cell nuclei were counterstained with Hoechst (blue). Percentage of cells with cloning ability (20 cells after 10 days). Colonies were categorized into large (>501 cells), medium (201–500 cells) and small (20–200 cells) size. All bars represent mean ± SD from three independent samples. **(B) Phenotypical characterization of ECFC.** Phase contrast micrographs from confluent monolayer of ECFCs with characteristic cobble-stone morphology. Indirect immunofluorescence of ECFCs showed positive staining for CD31, and vWF (both in green). Cell nuclei were counterstained with Hoechst (blue). Representative phase contrast micrographs of capillary-like tube formation by ECFCs on 2D-Matrigel. **(C) Representative phase contrast micrographs depicting the closure of a gap created in an ECFC monolayer**. Gap closure was monitored in response to FGF-2 (1 ng/mL) (E). Migratory capacity of ECFC in response to FGF-2 expressed as percentage of gap closure after 6 hours. Bars represent mean ± SE (n=3). We use the basal medium as a control.

Supplementary Figure S2

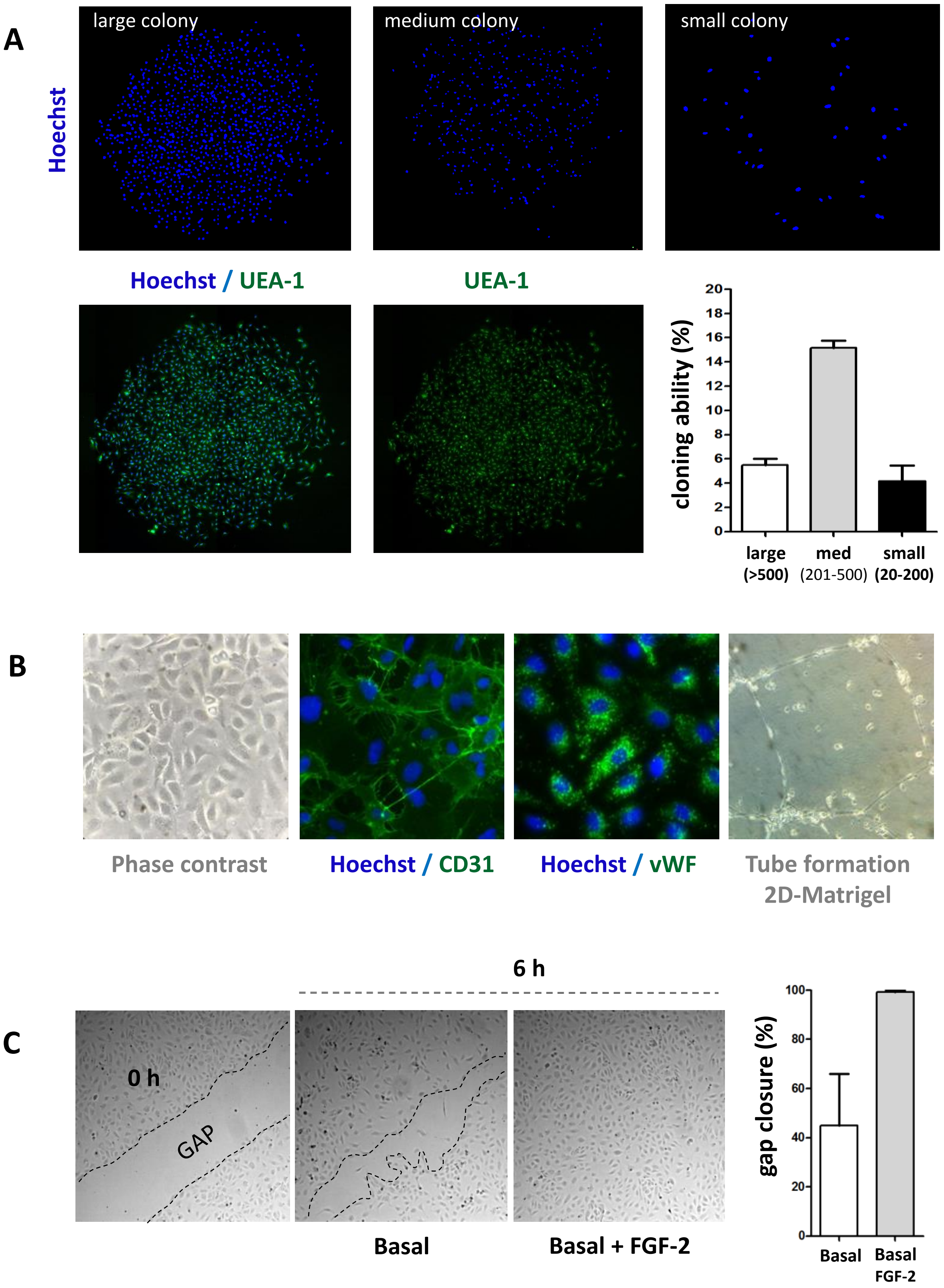

Supplementary Table S1. Quantitative comparison of secretome expression of healthy CAC (CAC control) and CAC treated with supernatants of atherosclerotic plaques (CAC+AP). Samples were run in triplicates. The table includes (from left to right): Uniprot accession number; protein description with gene name (GN);
Involved in (DAVID): MD: Macular degeneration; **AT:** Atherosclerosis; **HT**: Hypertension; **MI**: Myocardial Infarction; **CD**: Coronary disease; **DII**: Type II Diabetes; **CAD**: Coronary artery disease; **NV-MD**: Neovascularization and macular degeneration; **CNV**: Choroidal neovascularization; **CVD**: Cardiovascular disease; **AAA**: Abdominal aortic aneurysm; **HD-IS**: Heart disease ischemia; **VTB**: Venous thromboembolism; **MIS**: myocardial ischemia; **BH**: Brain hemorrhage; **TB**: Thrombosis; **DII-ED**: Type II diabetes and edema;
Involved in (String): RA: Regulation of angiogenesis; **RECM**: Regulation of endothelial cell migration; **A**: Angiogenesis; **RECP**: Regulation of endothelial cell proliferation; **RECC**: Regulation of endothelial cell chemotaxis; **PRA**: Positive regulation of angiogénesis; **AD:** Artery development; **RBVECM**: Regulation of blood vessel endothelial cell migration; **RVEGFRSP**: regulation of vascular endothelial growth factor receptor signaling pathway; **NRA**: Negative regulation of angiogenesis; **NRECM**: Negative regulation of endothelial cell migration; **PRECAP**: Positive regulation of endothelial cell apoptotic process; **NRECP**: Negative regulation of endothelial cell proliferation; **NRBVECM**: Negative regulation of blood vessel endothelial cell migration; **PRECM**: Positive regulation of endothelial cell migration; **PRECC**: Positive regulation of endothelial cell chemotaxis
Involved in (IPA): DV: Development of vasculature; **V:** Vasculogenesis; **A:** Angiogenesis; **N:** Necrosis; **AP:** Apoptosis; **AT:** Atherosclerosis lesion; **PVD:** Peripheral vascular disease.
Changes detected: protein up- or down-regulation (considering pvalue<0.05 and fold>1.5 in at least 2 out of 3 samples);
AP_C:-Log Student's T-test p-value AP_Ctr; AP_C:log2(AP_C); AP_C:Student's T-test Difference AP_Ctr

Accesion	Description	Involved in (DAVID)	Involved in (String)	Involved in (IPA)	changes detected	AP_C: -Log Student's T-test p-value AP_Ctr	AP_C: log2(AP/C)	AP_C: Student's T-test Difference AP_Ctr
**P35749**	Myosin-11 [MYH11]			DV, N, AP	Up-reg	3,94071	7,71909	7,13285
**P35580**	Myosin-10 [MYH10]		AD	DV, V, A, N, AP	Up-reg	3,1082	7,32816	6,65962
**P13611**	Versican core protein [VCAN]	AAA, BH		N, AP, AT	Up-reg	4,79669	7,02194	7,75568
**P09493**	Tropomyosin alpha-1 chain [TPM1]	CVD		N, AP	Up-reg	6,3478	6,61039	6,60877
**P98095**	Fibulin-2 [FBLN2]			DV, A	Up-reg	7,55087	6,57313	6,50101
**P23527**	Histone H2B type 1-O [HIST1H2BO]			N	Up-reg	1,54762	6,51005	4,38873
**Q05682**	Caldesmon [CALD1]				Up-reg	6,33948	6,45531	6,61368
**P98160**	Basement membrane-specific heparan sulfate proteoglycan core protein;Endorepellin;LG3 peptide [HSPG2]	AAA	RA, A, NRA	DV, V, A, AP, AT	Up-reg	3,86108	6,26962	7,07437
**Q15063**	Periostin [POSTN]			N	Up-reg	3,41778	6,193	6,43687
**P00734**	Prothrombin [F2]	AT, HT, MI, CD, DII, CAD, NV-MD, VTB, MIS, TB		DV, V, A, N, AP, PVD	Up-reg	4,59251	5,56742	5,62746
**Q16270**	Insulin-like growth factor-binding protein 7			DV, V, A, N, AP	Up-reg	4,94055	5,39443	5,30686

	[IGFBP7]							
**P08294**	Extracellular superoxide dismutase [Cu-Zn] [SOD3]	AT, HT, DII		N, AP	Up-reg	5,67541	5,33588	5,44871
**P05452**	Tetranectin [CLEC3B]				Up-reg	3,20395	5,32514	4,55342
**P08519**	Apolipoprotein(a) [LPA]	AT, MI, CD, DII, CAD, VTB		AT	Up-reg	3,51525	5,27458	5,77116
**P02763**	Alpha-1-acid glycoprotein 1 [ORM1]	TB		DV, V, A	Up-reg	2,43281	5,03809	4,35803
**P39060**	Collagen alpha-1(XVIII) chain; Endostatin [COL18A1]		A, PRECAP	DV, V, A, N, AP, AT	Up-reg	5,83644	5,02971	5,20378
**Q12805**	EGF-containing fibulin-like extracellular matrix protein 1 [EFEMP1]	MD, AT, NV-MD		N, AP	Up-reg	3,10514	4,84334	6,09534
**P01591**	Immunoglobulin J chain [IGJ]				Up-reg	3,0193	4,81263	4,38235
**P51888**	Prolargin [PRELP]	HT			Up-reg	2,31832	4,75712	3,85749
**P21980**	Protein-glutamine gamma-glutamyltransferase 2 [TGM2]			DV, V, A, N, AP	Up-reg	5,77687	4,60311	4,56404
**Q8N2S1**	Latent-transforming growth factor beta-binding protein 4 [LTBP4]	AAA			Up-reg	2,99213	4,40798	4,09782
**P02766**	Transthyretin [TTR]	CVD		N, AP	Up-reg	5,37588	4,36747	4,31563
**Q9UBX5**	Fibulin-5 [FBLN5]	MD, AT		DV, V, A	Up-reg	6,08199	4,36478	4,2932
**P35555**	Fibrillin-1 [FBN1]	CVD		DV, AP	Up-reg	2,8177	4,29115	4,1073
**Q01995**	Transgelin [TAGLN]				Up-reg	1,77199	4,28785	3,68712
**P21810**	Biglycan [BGN]			N, AP	Up-reg	3,33286	4,16016	4,35765
**P55290**	Cadherin-13 [CDH13]	HT, MI, CD, CAD	A, RECP	DV, V, A	Up-reg	4,55579	4,12511	4,3219
**Q14767**	Latent-transforming growth factor beta-binding protein 2 [LTBP2]	HT			Up-reg	4,02251	4,02096	4,66344
**Q8N1B4**	Vacuolar protein sorting-associated protein 52 homolog [VPS52]				Up-reg	2,47272	3,99467	3,71678
**Q99715**	Collagen alpha-1(XII) chain [COL12A1]				Up-reg	1,9382	3,98783	3,42282
**P19823**	Inter-alpha-trypsin inhibitor heavy chain H2 [ITIH2]				Up-reg	3,39344	3,92032	4,42653
**P35442**	Thrombospondin-2 [THBS2]	AT, MI, DII, HD-IS	RA, NRA	DV, V, A, N, AP, PVD	Up-reg	3,92497	3,79769	4,14596
**P20774**	Mimecan [OGN]				Up-reg	2,94287	3,74539	3,98849
**P80362**	Ig kappa chain V-I region WAT				Up-reg	5,50711	3,73266	3,7335
**P12111**	Collagen alpha-3(VI) chain [COL6A3]	HT			Up-reg	1,63946	3,71811	3,37314

**Q8IUX7**	Adipocyte enhancer-binding protein 1 [AEBP1]				Up-reg	2,54019	3,66254	4,1153
**P07360**	Complement component C8 gamma chain [C8G]				Up-reg	5,51186	3,58036	3,62757
**Q9NR12**	PDZ and LIM domain protein 7 [PDLIM7]			N, AP	Up-reg	5,02013	3,57052	3,56527
**P23142**	Fibulin-1 [FBLN1]			DV, V, A, N, AP	Up-reg	2,83917	3,52907	4,32393
**P01877**	Ig alpha-2 chain C region [IGHA2]				Up-reg	3,1107	3,50488	3,33492
**Q92743**	Serine protease HTRA1 [HTRA1]	MD, AT, NV-MD, CNV		DV, V, A, N, AP, PVD	Up-reg	2,25455	3,35251	2,95276
**P07357**	Complement component C8 alpha chain [C8A]	MD			Up-reg	2,75251	3,33309	3,34951
**P19827**	Inter-alpha-trypsin inhibitor heavy chain H1 [ITIH1]				Up-reg	2,74122	3,2537	4,12734
**P36955**	Pigment epithelium-derived factor [SERPINF1]	MD, NV-MD	RA, RECM, NRA, NRECM	DV, V, A, N, AP	Up-reg	3,02739	3,25342	3,76382
**Q05707**	Collagen alpha-1(XIV) chain [COL14A1]	CAD			Up-reg	2,15843	3,2461	4,05636
**P01871**	Ig mu chain C region [IGHM]			DV, N, AP	Up-reg	1,94498	3,18014	3,92311
**P02749**	Beta-2-glycoprotein 1 [APOH]	BH	RA, RECM, RECP, NRA, NRECM, NRECP	DV, V, A, PVD	Up-reg	2,35346	3,13551	3,86918
**P01623**	Ig kappa chain V-III region WOL				Up-reg	2,38652	3,0875	3,8975
**P04217**	Alpha-1B-glycoprotein [A1BG]				Up-reg	3,15997	3,06388	3,7646
**P02748**	Complement component C9 [C9]	MD		N	Up-reg	1,71671	3,01256	2,50894
**Q14766**	Latent-transforming growth factor beta-binding protein 1 [LTBP1]	AAA	AD	DV, V, A, N, AP	Up-reg	3,22071	2,91756	3,64212
**P13671**	Complement component C6 [C6]	MD	RA, PRA	DV, V, A, AP	Up-reg	2,447	2,85754	2,70732
**P01834**	Ig kappa chain C region [IGKC]				Up-reg	2,76872	2,84518	2,97599
**Q9GZM7**	Tubulointerstitial nephritis antigen-like [TINAGL1]			PVD	Up-reg	3,74772	2,74449	2,85028
**P39059**	Collagen alpha-1(XV) chain;Restin [COL15A1]		A	DV, V, A	Up-reg	2,52227	2,72633	2,54905
**P04004**	Vitronectin [VTN]	MD	RVEGFRSP	DV, V, A, N, AP	Up-reg	2,3212	2,71561	3,50268
**P22105**	Tenascin-X [TNXB]				Up-reg	2,24045	2,71004	3,36791
**P01861**	Ig gamma-4 chain C region [IGHG4]				Up-reg	3,03993	2,66387	3,29399
**P02760**	Protein AMBP;Alpha-1-microglobulin;Trypstatin [AMBP]				Up-reg	2,66783	2,63625	4,11806

**P13987**	CD59 glycoprotein [CD59]	MD		N, AP, AT	Up-reg	2,11873	2,57727	2,62439
**P01859**	Ig gamma-2 chain C region [IGHG2]				Up-reg	1,83008	2,56303	4,11973
**Q9Y6C2**	EMILIN-1 [EMILIN1]	HT	RA, RVEGFRSP, NRA	DV, A	Up-reg	2,24245	2,52992	2,27858
**P24821**	Tenascin [TNC]			DV, V, A, N, AP	Up-reg	2,03385	2,5264	4,2342
**P51884**	Lumican [LUM]			DV, N, AP	Up-reg	2,02112	2,52553	3,94152
**P01598**	Ig kappa chain V-I region EU				Up-reg	5,60493	2,50262	2,46254
**Q15113**	Procollagen C-endopeptidase enhancer 1 [PCOLCE]				Up-reg	2,05456	2,47702	3,2582
**P01857**	Ig gamma-1 chain C region [IGHG1]			DV, V, A, N, AP, AT	Up-reg	1,5969	2,47487	3,77513
**P01042**	Kininogen-1 [KNG1]	HT		DV, V, A, N, AP	Up-reg	1,97988	2,45579	2,59569
**P22352**	Glutathione peroxidase 3 [GPX3]	AT		PVD	Up-reg	2,14034	2,36639	2,18995
**O43866**	CD5 antigen-like [CD5L]			N, AP	Up-reg	1,42872	2,33294	2,69936
**P04003**	C4b-binding protein alpha chain [C4BPA]	MD			Up-reg	1,88068	2,28399	3,30807
**P18206**	Vinculin [VCL]			AP	Up-reg	2,08589	2,28272	1,98378
**P10643**	Complement component C7 [C7]	MD		N	Up-reg	2,60943	2,25598	2,21443
**P10909**	Clusterin;Clusterin beta chain;Clusterin alpha chain [CLU]	MD		DV, N, AP, PVD	Up-reg	1,66889	2,21434	4,31718
**P01024**	Complement C3 [C3]	MD, AT, CD, CNV, VTB	RA, PRA	DV, V, A, N, AP	Up-reg	1,56159	2,20624	4,31121
**P0CG05**	Ig lambda-2 chain C regions [IGLC2]				Up-reg	2,0969	2,20031	3,22741
**P27918**	Properdin [CFP]				Up-reg	2,64245	2,09854	2,72293
**P07996**	Thrombospondin-1 [THBS1]	AT, MI	RA, RECM, A, RECP, RECC, PRA, RBVECM, NRA, NRECM, PRECAP, NRECP, NRBVECM	DV, V, A, N, AP, AT, PVD	Up-reg	5,14234	2,03757	2,08905
**P12109**	Collagen alpha-1(VI) chain [COL6A1]			N, PVD	Up-reg	1,79444	1,96944	1,74301
**P08603**	Complement factor H [CFH]	MD, AT, HT, MI, CD, CAD, NV-MD, CNV, HD-IS		DV, N, AP, PVD	Up-reg	1,52351	1,96048	4,81755
**P02751**	Fibronectin;Anastellin [FN1]	MI, CAD, HD-IS	A	DV, V, A, N, AP, AT, PVD	Up-reg	1,53176	1,86957	4,08379
**P30626**	Sorcin [SRI]			N, AP	Up-reg	1,88707	1,86935	2,21259

**Q96PD5**	N-acetylmuramoyl-L-alanine amidase [PGLYRP2]				Up-reg	1,31346	1,85426	1,78891
**P02790**	Hemopexin [HPX]				Up-reg	1,45589	1,82852	3,38286
**P68032**	Actin, alpha cardiac muscle 1 [ACTC1;ACTA1]	AT			Up-reg	1,71959	1,73749	2,19259
**P02647**	Apolipoprotein A-I;Proapolipoprotein A-I;Truncated apolipoprotein A-I [APOA1]	AT, HT, MI, CD, DII, CAD, CVD, MIS		DV, V, A, N, AP, AT, PVD	Up-reg	1,30067	1,71092	3,67362
**P02649**	Apolipoprotein E [APOE]	MD, AT, HT, MI, CD, DII, CAD, CVD, HD-IS, VTB, MIS, BH, TB	RECM, RECP, AD, RBVECM, NRECM, NRECP, NRBVECM	DV, V, A, N, AP, AT, PVD	Up-reg	2,06689	1,66861	1,62544
**P00751**	Complement factor B [CFB]	MD, CD, DII, NV-MD, CNV		DV, V, A	Up-reg	1,35963	1,64395	2,68609
**P10412**	Histone H1.4 [HIST1H1E]				Up-reg	2,55638	1,58834	1,69159
**P02545**	Prelamin-A/C [LMNA]	AT, DII		N, AP	Up-reg	1,33029	1,56473	1,28913
**Q06828**	Fibromodulin [FMOD]	HT		DV, N, AP	Up-reg	2,07132	1,41227	1,58552
**P04792**	Heat shock protein beta-1 [HSPB1]		RA, RECM, RECC, PARA, RBVECM	DV, A, N, AP, PVD	Up-reg	1,37688	1,36198	2,53005
**Q96C19**	EF-hand domain-containing protein D2 [EFHD2]				Down-reg	1,82092	-1,35279	-1,56248
**P38606**	V-type proton ATPase catalytic subunit A [ATP6V1A]				Down-reg	2,3564	-1,36788	-1,46909
**P68036**	Ubiquitin-conjugating enzyme E2 L3 [UBE2L3]			N	Down-reg	2,05355	-1,3685	-1,39457
**Q8NC51**	Plasminogen activator inhibitor 1 RNA-binding protein [SERBP1]				Down-reg	1,4266	-1,37028	-1,853
**P13804**	Electron transfer flavoprotein subunit alpha, mitochondrial [ETFA]				Down-reg	2,08212	-1,37874	-1,47456
**P62333**	26S protease regulatory subunit 10B [PSMC6]			N	Down-reg	1,80978	-1,38455	-1,7583
**P46778**	60S ribosomal protein L21 [RPL21]				Down-reg	1,49301	-1,38506	-1,78494
**P07384**	Calpain-1 catalytic subunit [CAPN1]			DV, V, A, N, AP, PVD	Down-reg	2,05424	-1,38779	-1,519
**Q9Y3Z3**	Deoxynucleoside triphosphate triphosphohydrolase SAMHD1 [SAMHD1]				Down-reg	1,36825	-1,39775	-1,68385
**P05120**	Plasminogen activator inhibitor 2 [SERPINB2]	DII-ED		N	Down-reg	3,84344	-1,39834	-1,43621
**P46926**	Glucosamine-6-phosphate isomerase 1 [GNPDA1]				Down-reg	2,31969	-1,4037	-1,58169
**P48735**	Isocitrate dehydrogenase [NADP], mitochondrial [IDH2]			N	Down-reg	1,59202	-1,40477	-1,69282
**Q13596**	Sorting nexin-1 [SNX1]			N, AP	Down-reg	1,45031	-1,42052	-1,89752

**P06744**	Glucose-6-phosphate isomerase [GPI]		PRECM	N, AP	Down-reg	3,30528	-1,42199	-1,44301
**P51572**	B-cell receptor-associated protein 31 [BCAP31]			N, AP	Down-reg	1,64892	-1,42289	-1,69087
**Q15907**	Ras-related protein Rab-11B [RAB11B;RAB11A]				Down-reg	1,62331	-1,42462	-1,93442
**P05387**	60S acidic ribosomal protein P2 [RPLP2]				Down-reg	2,10566	-1,42581	-1,52105
**P11413**	Glucose-6-phosphate 1-dehydrogenase [G6PD]	DII-ED		DV, V, A, N, AP	Down-reg	1,75456	-1,43054	-1,54701
**P09622**	Dihydrolipoyl dehydrogenase, mitochondrial [DLD]				Down-reg	2,42723	-1,44192	-1,48184
**Q9ULZ3**	Apoptosis-associated speck-like protein containing a CARD [PYCARD]			N, AP	Down-reg	1,70653	-1,44324	-1,67997
**P17987**	T-complex protein 1 subunit alpha [TCP1]	DII-ED		N, AP	Down-reg	1,73033	-1,4445	-1,73961
**O75368**	SH3 domain-binding glutamic acid-rich-like protein [SH3BGRL]				Down-reg	2,06454	-1,45478	-1,60867
**P22897**	Macrophage mannose receptor 1 [MRC1]				Down-reg	1,63366	-1,48626	-1,65647
**Q9Y2J2**	Band 4.1-like protein 3;Band 4.1-like protein 3, N-terminally processed [EPB41L3]				Down-reg	1,50804	-1,49585	-1,96079
**P20810**	Calpastatin [CAST]	DII-ED		N, AP	Down-reg	2,12564	-1,49921	-1,67422
**P62277**	40S ribosomal protein S13 [RPS13]			N	Down-reg	1,97026	-1,50679	-1,67412
**P05141**	ADP/ATP translocase 2 [SLC25A5;SLC25A4]				Down-reg	2,32393	-1,51076	-1,58079
**P12955**	Xaa-Pro dipeptidase [PEPD]				Down-reg	3,99925	-1,52204	-1,56029
**P05388**	60S acidic ribosomal protein P0 [RPLP0;RPLP0P6]				Down-reg	1,92405	-1,53389	-1,36861
**P00491**	Purine nucleoside phosphorylase [PNP]			N, AP	Down-reg	1,6357	-1,53418	-1,76698
**Q9NRV9**	Heme-binding protein 1 [HEBP1]	DII-ED			Down-reg	1,70471	-1,54247	-1,9522
**Q9P2E9**	Ribosome-binding protein 1 [RRBP1]			AP, PVD	Down-reg	1,32535	-1,54749	-1,65367
**Q7L576**	Cytoplasmic FMR1-interacting protein 1 [CYFIP1;CYFIP2]				Down-reg	2,55962	-1,5503	-1,67396
**Q13177**	Serine/threonine-protein kinase PAK 2 [PAK2;PAK3]				Down-reg	2,83287	-1,55317	-1,69814
**P31949**	Protein S100-A11 [S100A11]			N, AP	Down-reg	2,572	-1,55954	-1,65173
**P40227**	T-complex protein 1 subunit zeta [CCT6A]			N	Down-reg	2,34308	-1,56355	-1,69742
**P49207**	60S ribosomal protein L34 [RPL34]			N	Down-reg	2,36568	-1,57484	-1,68979
**Q9H0U4**	Ras-related protein Rab-1B [RAB1B;RAB1C]				Down-reg	2,0209	-1,58128	-1,85923

**P43490**	Nicotinamide phosphoribosyltransferase [NAMPT]	DII-ED		N, AP	Down-reg	5,5009	-1,59641	-1,60329
**P62750**	60S ribosomal protein L23a [RPL23A]				Down-reg	1,36639	-1,62725	-2,233
**P23368**	NAD-dependent malic enzyme, mitochondrial [ME2]				Down-reg	2,51691	-1,63148	-1,72173
**P38159**	RNA-binding motif protein, X chromosome [RBMX;RBMXL1]				Down-reg	1,64196	-1,63702	-2,23973
**P04899**	Guanine nucleotide-binding protein G(i) subunit alpha-2 [GNAI2]	DII-ED		N, AP	Down-reg	1,51477	-1,64588	-1,98609
**P99999**	Cytochrome c [CYCS]			DV, V, A, N, AP	Down-reg	1,77605	-1,6564	-1,83615
**Q09666**	Neuroblast differentiation-associated protein AHNAK [AHNAK]				Down-reg	3,04287	-1,66397	-1,6404
**Q07020**	60S ribosomal protein L18 [RPL18]	DII-ED			Down-reg	1,59932	-1,67484	-2,60828
**Q99538**	Legumain [LGMN]		PRECM, PRECC	N, AP	Down-reg	1,67923	-1,67935	-1,62799
**P09601**	Heme oxygenase 1 [HMOX1]	DII-ED	PRECM	DV, V, A, N, AP, AT	Down-reg	2,30319	-1,68142	-1,76302
**P00915**	Carbonic anhydrase 1 [CA1]			PVD	Down-reg	2,23421	-1,71386	-1,47567
**O94973**	AP-2 complex subunit alpha-2 [AP2A2]			N, AP	Down-reg	2,36841	-1,71875	-1,79744
**P48506**	Glutamate--cysteine ligase catalytic subunit [GCLC]			DV, V, A, N, AP	Down-reg	2,18445	-1,72473	-1,75925
**P20618**	Proteasome subunit beta type-1 [PSMB1]			N, AP	Down-reg	1,67282	-1,73787	-2,23424
**P05107**	Integrin beta-2 [ITGB2]	DII-ED		DV, V, A, N, AP	Down-reg	1,43437	-1,74988	-2,82013
**O75396**	Vesicle-trafficking protein SEC22b [SEC22B]				Down-reg	2,04251	-1,75399	-2,08281
**P22102**	Trifunctional purine biosynthetic protein adenosine-3 [GART]				Down-reg	3,76937	-1,76718	-1,81265
**P27635**	60S ribosomal protein L10 [RPL10]	DII-ED		N, AP	Down-reg	2,09631	-1,78761	-2,09582
**Q13765**	Nascent polypeptide-associated complex subunit alpha[NACA]				Down-reg	2,45714	-1,7887	-2,04725
**P54577**	Tyrosine--tRNA ligase, cytoplasmic;Tyrosine--tRNA ligase, cytoplasmic, N-terminally processed [YARS]			DV, V, A, N, AP	Down-reg	4,71201	-1,79676	-1,82519
**P29350**	Tyrosine-protein phosphatase non-receptor type 6 [PTPN6]			DV, V, A, N, AP	Down-reg	2,98869	-1,81359	-1,95051
**P57737**	Coronin-7 [CORO7]				Down-reg	2,25326	-1,82253	-2,07896
**P49411**	Elongation factor Tu, mitochondrial [TUFM]			N	Down-reg	1,88585	-1,82583	-2,21553
**Q14204**	Cytoplasmic dynein 1 heavy chain 1 [DYNC1H1]			N, AP	Down-reg	1,86789	-1,83118	-2,44281

**P30740**	Leukocyte elastase inhibitor [SERPINB1]			AP	Down-reg	2,86062	-1,83651	-1,85937
**P04080**	Cystatin-B [CSTB]	DII-ED		N, AP	Down-reg	1,71761	-1,85654	-2,209
**O15400**	Syntaxin-7 [STX7]			DV, V, A	Down-reg	3,06699	-1,85937	-1,92248
**P11766**	Alcohol dehydrogenase class-3 [ADH5]			N, AP	Down-reg	2,35907	-1,88555	-2,37594
**P30046**	D-dopachrome decarboxylase;D-dopachrome decarboxylase-like protein [DDT;DDTL]	DII-ED			Down-reg	3,87656	-1,90986	-1,95081
**P62979**	Ubiquitin-40S ribosomal protein S27a [RPS27A;UBA52;UBB;UBC]	DII-ED			Down-reg	2,44263	-1,91284	-1,76344
**P61254**	60S ribosomal protein L26;60S ribosomal protein L26-like 1 [RPL26;RPL26L1]				Down-reg	2,11036	-1,91526	-2,35411
**P63000**	Ras-related C3 botulinum toxin substrate 1 [RAC1;RAC3]				Down-reg	2,58021	-1,91988	-2,1965
**P62328**	Thymosin beta-4 [TMSB4X]		PRECM, PRECC	N, AP	Down-reg	2,35961	-1,95009	-1,97773
**Q8TD55**	Pleckstrin homology domain-containing family O member 2 [PLEKHO2]			PVD	Down-reg	2,1621	-1,9704	-2,50221
**Q10567**	AP-1 complex subunit beta-1 [AP1B1]				Down-reg	3,44594	-1,97308	-2,0932
**Q02818**	Nucleobindin-1 [NUCB1]				Down-reg	2,36298	-1,98561	-2,56096
**P08575**	Receptor-type tyrosine-protein phosphatase C [PTPRC]			N, AP	Down-reg	1,5796	-2,03222	-1,86929
**P07900**	Heat shock protein HSP 90-alpha [HSP90AA1]	DII-ED		DV, A, N, AP	Down-reg	3,50808	-2,04896	-2,09066
**P31948**	Stress-induced-phosphoprotein 1 [STIP1]			N, AP, PVD	Down-reg	2,06037	-2,07716	-2,53916
**Q8WZ42**	Titin [TTN]	DII-ED		N, AP	Down-reg	2,74687	-2,15207	-2,30585
**O15144**	Actin-related protein 2/3 complex subunit 2 [ARPC2]				Down-reg	2,69524	-2,15733	-2,17038
**P48643**	T-complex protein 1 subunit epsilon [CCT5]			N	Down-reg	2,10431	-2,15874	-2,74118
**P27824**	Calnexin [CANX]	DII-ED		AP	Down-reg	3,22914	-2,17833	-2,20474
**P22234**	Multifunctional protein ADE2 [PAICS]				Down-reg	3,07392	-2,1816	-2,3894
**P63244**	Guanine nucleotide-binding protein subunit beta-2-like 1 [GNB2L1]			N, AP	Down-reg	2,44066	-2,22585	-2,25096
**Q14764**	Major vault protein [MVP]			N, AP	Down-reg	2,66291	-2,22658	-2,39029
**P07948**	Tyrosine-protein kinase Lyn [LYN]			N, AP, PVD	Down-reg	3,69766	-2,24575	-2,34153
**P29966**	Myristoylated alanine-rich C-kinase substrate [MARCKS]			PVD	Down-reg	2,57345	-2,28512	-2,31326

**P35754**	Glutaredoxin-1 [GLRX]		DV, V, A, N, AP, PVD	Down-reg	3,0061	-2,28758	-2,51191
**Q16851**	UTP--glucose-1-phosphate uridylyltransferase [UGP2]			Down-reg	1,99549	-2,30258	-2,60516
**P48444**	Coatomer subunit delta [ARCN1]		N	Down-reg	3,29111	-2,34547	-2,59126
**P00352**	Retinal dehydrogenase 1 [ALDH1A1]		N, AP	Down-reg	2,79786	-2,36968	-2,51627
**Q00610**	Clathrin heavy chain 1 [CLTC]	DII-ED	N, AP	Down-reg	3,20013	-2,38527	-2,60164
**P80723**	Brain acid soluble protein 1 [BASP1]		AP	Down-reg	2,08773	-2,40163	-2,91805
**P08238**	Heat shock protein HSP 90-beta [HSP90AB1]	DII-ED	N, AP	Down-reg	2,76504	-2,44728	-2,62333
**Q9UJ70**	N-acetyl-D-glucosamine kinase [NAGK]			Down-reg	3,43288	-2,58085	-2,82621
**Q96CW1**	AP-2 complex subunit mu [AP2M1]		N, AP	Down-reg	3,23713	-2,58161	-2,80052
**P02730**	Band 3 anion transport protein [SLC4A1]	DII-ED		Down-reg	3,32109	-2,61667	-2,75014
**O00244**	Copper transport protein ATOX1 [ATOX1]		AP	Down-reg	2,95074	-2,63457	-3,11532
**P07108**	Acyl-CoA-binding protein [DBI]			Down-reg	4,61842	-2,67264	-2,74791
**P23284**	Peptidyl-prolyl cis-trans isomerase B [PPIB]			Down-reg	2,11906	-2,77179	-2,63302
**P32969**	60S ribosomal protein L9 [RPL9]		N, PVD	Down-reg	4,47439	-2,93634	-3,0413
**P51659**	Peroxisomal multifunctional enzyme type 2 [HSD17B4]	DII-ED		Down-reg	3,84746	-3,96407	-3,94417
**P62191**	26S protease regulatory subunit 4 [PSMC1]		N	Down-reg	5,37239	-4,49194	-4,67209

Supplementary Table S2. Functional and disease classification of proteins differentially released by CAC in response to the incubation with atherosclerotic factors. Protein classification was made with the IPA software, based on biomedical literature and integrated databases. The table includes the main categories in which differential proteins are involved, related functions or diseases, predicted activation or inhibition for these functions, number of molecules per category identified in the AP secretome, the p-values assigned and the gene names. The p-value provided by IPA is calculated using the right-tailed Fisher Exact Test, and it represents the likelihood that the association between a set of focus genes in your experiment and a given process or pathway is due to random chance. The smaller the p-value the less likely that the association is random and the more significant the association. P-values <0.05 are statistically significant. Functions discussed within the min manuscript are shown in **bold letters**. Legend: ↑ up-regulated; ↓ down-regulated

Categories	Disease or Functions Annotation	Predicted	Molecules	p-value	Molecules
Cardiovascular System Development	Cell movement of endothelial cell lines	Inhibition	12	$1.12E^{-9}$	↑CDH13, ↑FN1, ↑VTN, ↑SERPINF1, ↑KNG1, ↑MYH11, ↑TNC, ↑IGHG1, ↑APOE, ↑THBS1, ↑COL18A1, ↓YARS
	**Cell movement of EC**	**Inhibition**	**20**	$\mathbf{1.01E^{-8}}$	**↑CDH13, ↑SERPINF1, ↑APOH, ↑THBS1, ↑COL18A1, ↑HSPB1, ↑FN1, ↑VTN, ↑ORM1, ↑THBS2, ↑KNG1, ↑HSPG2, ↑APOE, ↓G6PD, ↓ITGB2, ↓PTPN6, ↓HSP90AB1, ↓GPI, ↓TMSB10/TMSB4X, ↓MARCKS**
	Development of vasculature	Inhibition	48	$1.58E^{-15}$	↑FBLN2, ↑CFB, ↑C3, ↑EMILIN1, ↑SERPINF1, ↑APOH, ↑HTRA1, ↑THBS1, ↑CFH, ↑FN1, ↑FBLN5, ↑KNG1, ↑MYH11, ↑IGHM, ↑TNC, ↑IGHG1, ↑F2, ↑CDH13, ↑FBN1, ↑COL15A1, ↑MYH10, ↑C6, ↑COL18A1, ↑HSPB1, ↑FBLN1, ↑VTN, ↑IGFBP7, ↑CLU, ↑ORM1, ↑THBS2, ↑APOA1, ↑HSPG2, ↑APOE, ↑LUM, ↑FMOD, ↑TGM2, ↑LTBP1, ↓YARS, ↓ITGB2, ↓CYCS, ↓PTPN6, ↓STX7, ↓HSP90AA1, ↓G6PD, ↓GCLC, ↓GLRX, ↓CAPN1, ↓HMOX1
	**EC development**	**Inhibition**	**16**	$\mathbf{4.54E^{-6}}$	**↑CDH13, ↑C3, ↑SERPINF1, ↑APOH, ↑THBS1, ↑COL18A1, ↑FN1, ↑VTN, ↑KNG1, ↑APOA1, ↑HSPG2, ↑IGHG1, ↑APOE, ↑F2, ↓G6PD, ↓HMOX1**
	**Migration of EC**	**Inhibition**	**18**	$\mathbf{7.64E^{-8}}$	**↑CDH13, ↑SERPINF1, ↑APOH, ↑THBS1, ↑COL18A1, ↑HSPB1, ↑FN1, ↑VTN, ↑ORM1, ↑KNG1, ↑APOE, ↓G6PD, ↓ITGB2, ↓PTPN6, ↓HSP90AB1, ↓GPI, ↓TMSB10/TMSB4X, ↓MARCKS**
	Morphology of blood vessel	---	22	$2.23E^{-11}$	↑FBN1, ↑COL15A1, ↑MYH10, ↑CFH, ↑FBLN1, ↑FBLN5, ↑FN1, ↑VTN, ↑CLU, ↑THBS2, ↑KNG1, ↑MYH11, ↑HSPG2, ↑APOE, ↑LUM, ↑FMOD, ↑LTBP1, ↓G6PD, ↓GCLC, ↓HMOX1
	**Vasculogenesis**	**Activation**	**37**	$\mathbf{4.10E^{-13}}$	**↑CFB, ↑C3, ↑SERPINF1, ↑APOH, ↑HTRA1, ↑THBS1, ↑FN1, ↑FBLN5, ↑KNG1, ↑TNC, ↑IGHG1, ↑F2, ↑CDH13, ↑COL15A1, ↑MYH10, ↑C6, ↑COL18A1, ↑FBLN1, ↑VTN, ↑IGFBP7, ↑ORM1, ↑THBS2, ↑APOA1, ↑HSPG2, ↑APOE, ↑TGM2, ↑LTBP1, ↓YARS, ↓ITGB2, ↓CYCS, ↓PTPN6, ↓STX7, ↓G6PD, ↓GCLC, ↓GLRX, ↓CAPN1, ↓HMOX1**
	Adhesion of EC	Activation	11	$2.21E^{-7}$	↑FBLN5, ↑FN1, ↑VTN, ↑IGFBP7, ↑THBS1, ↑COL18A1, ↑F2, ↓RACK1, ↓ITGB2,

					↓TMSB10/TMSB4X, ↓SLC4A1
	**Angiogenesis**	**Inhibition**	**41**	**$6.80E^{-13}$**	**↑FBLN2, ↑CFB, ↑C3, ↑EMILIN1, ↑SERPINF1, ↑APOH, ↑HTRA1, ↑THBS1, ↑FN1, ↑FBLN5, ↑KNG1, ↑TNC, ↑IGHG1, ↑F2, ↑CDH13, ↑COL15A1, ↑MYH10, ↑C6, ↑COL18A1, ↑HSPB1, ↑FBLN1, ↑VTN, ↑IGFBP7, ↑ORM1, ↑THBS2, ↑APOA1, ↑HSPG2, ↑APOE, ↑TGM2, ↑LTBP1, ↓YARS, ↓ITGB2, ↓CYCS, ↓PTPN6, ↓STX7, ↓HSP90AA1, ↓G6PD, ↓GCLC, ↓GLRX, ↓CAPN1, ↓HMOX1**
	Binding of EC	Activation	15	$1.36E^{-9}$	↑C3, ↑THBS1, ↑COL18A1, ↑LPA, ↑FBLN5, ↑FN1, ↑VTN, ↑IGFBP7, ↑KNG1, ↑F2, ↑BGN, ↓RACK1, ↓ITGB2, ↓TMSB10/TMSB4X, ↓SLC4A1
	**Binding of vascular EC**	**Inhibition**	**10**	**$4.51E^{-7}$**	**↑FN1, ↑VTN, ↑IGFBP7, ↑KNG1, ↑THBS1, ↑F2, ↑BGN, ↓RACK1, ↓ITGB2, ↓TMSB10/TMSB4X**
Cell death and survival	Cell survival	Inhibition	42	$1.95E^{-7}$	↑CD59, ↑C3, ↑VCL, ↑SERPINF1, ↑THBS1, ↑CFH, ↑LMNA, ↑FN1, ↑MYH11, ↑IGHM, ↑F2, ↑CDH13, ↑COL18A1, ↑HSPB1, ↑POSTN, ↑SOD3, ↑VTN, ↑IGFBP7, ↑CLU, ↑THBS2, ↑VCAN, ↑APOE, ↑TGM2, ↓ITGB2, ↓ADH5, ↓SERPINB2, ↓PRPN6, ↓UBE2L3, ↓GCLC, ↓GLRX, ↓RACK1, ↓CAPN1, ↓TCP1, ↓PPIB, ↓HMOX1, ↓PYCARD, ↓NAMPT, ↓PTPRC, ↓HSP90AB1, ↓BCAP31, ↓MVP, ↓LYN
	**Necrosis**	**Activation**	**92**	**$2.54E^{-23}$**	**↑C3, ↑SERPINF1, ↑HTRA1, ↑HIST1H2BO, ↑THBS1, ↑LMNA, ↑MYH11, ↑TNC, ↑IGHM, ↑F2, ↑C7, ↑HSPB1, ↑FBLN1, ↑SOD3, ↑PDLIM7, ↑CLU, ↑THBS2, ↑VCAN, ↑APOA1, ↑FMOD, ↑TGM2, ↑TTR, ↑CD59, ↑EFEMP1, ↑COL6A1, ↑SRI, ↑CFH, ↑FN1, ↑KNG1, ↑IGHG1, ↑C9, ↑TPM1, ↑CD5L, ↑MYH10, ↑COL18A1, ↑POSTN, ↑VTN, ↑IGFBP7, ↑APOE, ↑LUM, ↑BGN, ↑LTBP1, ↓YARS, ↓LGMN, ↓TUFM, ↓ITGB2, ↓CYCS, ↓RPS13, ↓ALDH1A1, ↓SERPINB2, ↓GPI, ↓UBE2L3, ↓DYNC1H1, ↓CLTC, ↓CSTB, ↓TCP1, ↓PSMC6, ↓IDH2, ↓LYN, ↓CCT6A, ↓STIP1, ↓PSMB1, ↓RPL9, ↓SNX1, ↓GNAI2, ↓ADH5, ↓PSMC1, ↓PNP, ↓PTPN6, ↓HSP90AA1, ↓CCT5, ↓S100A11, ↓G6PD, ↓GCLC, ↓GLRX, ↓RACK 1, ↓CAPN1, ↓HMOX1, ↓PYCARD, ↓NAMPT, ↓AP2A2, ↓CAST, ↓RPL34, ↓PTPRC, ↓AP2M1, ↓HSP90AB1, ↓TTN, ↓ARCN1, ↓BCAP31, ↓TMSB10/TMSB4X, ↓RPL10, ↓MVP**
	**Apoptosis**	**Inhibition**	**84**	**$2.10E^{-18}$**	**↑VCL, ↑C3, ↑SERPINF1, ↑HTRA1, ↑THBS1, ↑LMNA, ↑MYH11, ↑TNC, ↑IGHM, ↑F2, ↑FBN1, ↑C6, ↑HSPB1, ↑FBLN1, ↑SOD3, ↑PDLIM7, ↑CLU, ↑THBS2, ↑VCAN, ↑APOA1, ↑HSPG2, ↑FMOD, ↑TGM2, ↑TTR, ↑CD59, ↑EFEMP1, ↑SRI, ↑CFH, ↑FN1, ↑KNG1, ↑IGHG1, ↑TPM1, ↑CD5L, ↑MYH10, ↑COL18A1, ↑VTN, ↑IGFBP7, ↑APOE, ↑LUM, ↑BGN, ↑LTBP1, ↓YARS, ↓LGMN, ↓BASP1, ↓ITGB2, ↓CYCS, ↓ALDH1A1, ↓GPI, ↓DYNC1H1, ↓CLTC, ↓CSTB, ↓TCP1, ↓ATOX1,**

					↓**LYN,** ↓**SERPINB1,** ↓**STIP1,** ↓**PSMB1,** ↓**SNX1,** ↓**GNAI2,** ↓**ADH5,** ↓**CANX,** ↓**PNP,** ↓**PTPN6,** ↓**HSP90AA1,** ↓**S100A11,** ↓**G6PD,** ↓**GCLC,** ↓**GLRX,** ↓**RACK1,** ↓**CAPN1,** ↓**RRBP1,** ↓**HMOX1,** ↓**PYCARD,** ↓**NAMPT,** ↓**AP2A2,** ↓**CAST,** ↓**PTPRC,** ↓**AP2M1,** ↓**HSP90AB1,** ↓**TTN,** ↓**BCAP31,** ↓**TMSB10/TMSB4X,** ↓**RPL10,** ↓**MVP**
Cell to cell signaling and interaction	Adhesion of blood cells	Activation	29	$1.32E^{-16}$	↑CD59, ↑C3, ↑APOH, ↑THBS1, ↑CFH, ↑FN1, ↑KNG1, ↑IGHM, ↑TNC, ↑F2, ↑VTN, CLU, ↑ORM1, ↑THBS2, ↑APOA1, ↑C4BPA, ↑CFP, ↑HSPG2, ↑APOE, ↑TGM2, ↓ITGB2, ↓GNAI2, ↓MRC1, ↓PTPN6, ↓SLC4A1, ↓GLRX, ↓PPIB, ↓PTPRC, ↓LYN
	Aggregation of blood cells	Activation	18	$2.67E^{-11}$	↑C3, ↑SERPINF1, ↑THBS1, ↑CFH, ↑GPX3, ↑VTN, ↑CLU, ↑THBS2, ↑KNG1, ↑C4BPA, ↑IGHM, ↑F2, ↓CAPN1, ↓ITGB2, ↓GNAI2, ↓CAST, ↓PTPN6, ↓LYN
	Binding of blood cells	Activation	30	$4.71E^{-16}$	↑CD59, ↑C3, ↑APOH, ↑THBS1, ↑CFH, ↑LPA, ↑FN1, ↑KNG1, ↑IGHM, ↑TNC, F2, ↑VTN, ↑CLU, ↑ORM1, ↑THBS2, ↑APOA1, ↑C4BPA, ↑CFP, ↑HSPG2, ↑APOE, ↑TGM2, ↓ITGB2, ↓GNAI2, ↓MRC1, ↓PTPN6, ↓SLC4A1, ↓GLRX, ↓PPIB, ↓PTPRC, ↓LYN
	Phagocytosis by macrophages	Inhibition	9	$5.92E^{-6}$	↑CD5L, ↑VTN, ↑C2, ↑THBS1, ↑LUM, ↑TGM2, ↓ITGB2, ↓PTPRC, ↓HMOX1
	Phagocytosis of blood cells	Inhibition	13	$2.29E^{-8}$	↑CD5L, ↑C3, ↑THBS1, ↑CFH, ↑VTN, ↑APOA1, ↑LUM, ↑TGM2, ↓GLRX, ↓ITGB2, ↓HMOX1, ↓PTPRC, ↓LYN
	Activation of blood cells	Activation	30	$2.74E^{-9}$	↑CD59, ↑C3, ↑SERPINF1, ↑APOH, ↑THBS1, ↑CFH, ↑FN1, ↑KNG1, ↑IGHM, ↑TNC, ↑IGHG1, ↑F2, ↑C6, ↑VTN, ↑VCAN, ↑APOA1, ↑HSPG2, ↑APOE, ↑PGLYRP2, ↑TGM2, ↑LTBP1, ↓ITGB2, ↓GNAI2, ↓PTPN6, ↓HEBP1, ↓HMOX1, ↓PYCARD, ↓PTPRC, ↓AHNAK, ↓LYN
Cardiovascular Disease	**Atherosclerotic lesion**	**Inhibition**	**11**	$\mathbf{1.13E^{-6}}$	↑**LPA,** ↑**CD59,** ↑**FN1,** ↑**VCAN,** ↑**APOA1,** ↑**HSPG2,** ↑**IGHG1,** ↑**APOE,** ↑**THBS1,** ↑**COL18A1,** ↓**HMOX1**
	Familial cardiovascular disease	---	24	$8.27E^{-10}$	↑FBN1, ↑CFB, ↑C3, ↑VCL, ↑HTRA1, ↑THBS1, ↑CFH, ↑LMNA, ↑LPA, ↑GBLN1, ↑TINAGL1, ↑IGFBP7, ↑THBS2, ↑MYH11, ↑APOA1, ↑HSPG2, ↑APOE, ↑TPM1, ↑F2, ↑BGN, ↑LTBP1, ↑TTR, ↓GNAI2, ↓TTN
	Familial vascular disease	---	14	$2.12E^{-7}$	↑FBN1, ↑CFB, ↑C3, ↑HTRA1, ↑THBS1, ↑CFH, ↑TINAGL1, ↑IGFBP7, ↑THBS2, ↑MYH11, ↑HSPG2, ↑F2, ↑BGN, ↓LTBP1
	Peripheral vascular disease	Inhibition	23	$2.42E^{-9}$	↑COL6A1, ↑APOH, ↑HTRA1, ↑THBS1, ↑CFH, ↑LPA, ↑HSPB1, ↑FN1, ↑GPX3, ↑TINAGL1, ↑CLU, ↑THBS2, ↑APOA1, ↑APOE, ↑F2, ↓GLRX, ↓RRBP1, ↓STIP1, ↓PLEKHO2, ↓RPL9, ↓CA1, ↓MARCKS, ↓LYN
	Thrombus	---	14	$5.28E^{-8}$	↑FBN1, ↑CFB, ↑C3, ↑APOH, ↑THBS1, ↑CFH, ↑LPA, ↑GPX3, ↑VTN, ↑KNG1, ↑APOE, ↑F2, ↓CAPN1, ↓HMOX1

	Thrombosis of artery	---	5	$7.18E^{-6}$	↑APOH, ↑APOE, ↑THBS1, ↑F2, ↓CAPN1
	**Vascular lesion**	**Inhibition**	**16**	$\mathbf{7.59E^{-9}}$	**↑CD59, ↑FBN1, ↑THBS1, ↑COL18A1, ↑LPA, ↑FN1, ↑IGFBP7, ↑VCAN, ↑MYH11, ↑APOA1, ↑HSPG2, ↑IGHG1, ↑APOE, ↓RRBP1, ↓HMOX1, ↓PTPRC**
	Abnormal morphology of blood vessel	---	15	$1.13E^{-7}$	↑FBN1, ↑COL15A1, ↑CFH, ↑FBLN1, ↑FN1, ↑CLU, ↑THBS2, ↑MYH11, ↑HSPG2, ↑APOE, ↑LUM, ↑FMOD, ↑LTBP1, ↓G6PD, ↓HMOX1
Endocrine System Disorders	Diabetic complication	---	13	$2.50E^{-6}$	↑COL14A1, ↑COL15A1, ↑COL6A3, ↑SERPINF1, ↑COL6A1, ↑COL18A1, ↑COL12A1, ↑FN1, ↑CLU, ↑APOE, ↑CLEC3B, ↑AMBP, ↓CA1
	Diabetic foot ulcer disorder	---	6	$3.24E^{-6}$	↑COL12A1, ↑COL14A1, ↑COL15A1, ↑COL6A3, ↑COL6A1, ↑COL18A1
	Diabetes mellitus	---	39	$6.06E^{-10}$	↑CFB, ↑COL14A1, ↑C3, ↑SERPINF1, ↑COL6A1, ↑HIST1H2BO, ↑CFH, ↑LMNA, ↑LPA, ↑COL12A1, ↑FN1, ↑MYH11, ↑IGHM, ↑TNC, ↑IGHG1, ↑CLEC3B, ↑AMBP, ↑AEBP1, ↑COL15A1, ↑COL6A3, ↑COL18A1, ↑HSPB1, ↑IGFBP7, ↑THBS2, ↑CLU, ↑TNXB, ↑APOA1, ↑APOE, ↑TGM2, ↑TTR, ↓ITGB2, ↓PSMB1, ↓MRC1, ↓ALDH1A1, ↓CANX, ↓PTPRC, ↓PSMC6
Hematological system development and function	Coagulation of blood	Inhibition	17	$8.80E^{-9}$	↑CD59, ↑C3, ↑SERPINF1, ↑APOH, ↑THBS1, ↑VTN, ↑THBS2, ↑KNG1, ↑VCAN, ↑HSPG2, ↑APOE, ↑C9, ↑F2, ↓CAPN1, ↓GNAI2, ↓PTPN6, ↓LYN
	Hemostasis	Activation	23	$1.07E^{-12}$	↑CD59, ↑C3, ↑SERPINF1, ↑APOH, ↑THBS1, ↑LPA, ↑FN1, ↑VTN, ↑THBS2, ↑VCAN, ↑KNG1, ↑IGHM, ↑HSPG2, ↑APOE, ↑C9, ↑F2, ↓CAPN1, ↓PPIB, ↓GNAI2, ↓SERPINB2, ↓PTPN6, ↓GPI, ↓LYN
	Accumulation of blood cells	Inhibition	15	$3.94E^{-7}$	↑CD5L, ↑C3, ↑C6, ↑THBS1, ↑COL18A1, ↑POSTN, ↑APOA1, ↑IGHG1, ↑APOE, ↑F2, ↑BGN, ↓ITGB2, ↓HMOX1, ↓GNAI2, ↓LYN
Inflammatory response	Immune response of cells	Inhibition	32	$8.92E^{-14}$	↑CD59, ↑C3, ↑THBS1, ↑CFH, ↑FN1, ↑IGHA2, ↑IGHM, ↑IGHG1, ↑IGKC, ↑CD5L, ↑HSPB1, ↑POSTN, ↑VTN, ↑APOA1, ↑APOE, ↑LUM, ↑TGM2, ↑LTBP1, ↓ITGB2, ↓MRC1, ↓PNP, ↓PTPN6, ↓HSP90AA1, ↓UBE2L3, ↓GLRX, ↓RACK1, ↓CLTC, ↓HMOX1, ↓PYCARD, ↓NAMPT, ↓PTPRC, ↓LYN
	**Inflammatory response**	**Activation**	**38**	$\mathbf{9.27E^{-14}}$	**↑C3, ↑SERPINF1, ↑APOH, ↑THBS1, ↑CFH, ↑LPA, ↑FN1, ↑KNG1, ↑IGHM, ↑TNC, ↑IGHG1, ↑F2, ↑C6, ↑COL18A1, ↑HSPB1, ↑SOD3, ↑VTN, ↑ORM1, ↑THBS2, ↑APOA1, ↑HSPG2, ↑APOE, ↑PGLYRP2, ↑LUM, ↑TGM2, ↑LTBP1, ↓SERPINB1, ↓YARS, ↓LGMN, ↓ITGB2, ↓GNAI2, ↓PTPN6, ↓HEBP1, ↓PPIB, ↓HMOX1, ↓PYCARD, ↓TMSB10/TMSB4X, ↓LYN**

Supplementary Table S3. Statistical data (p-values) related to the evaluation of the effect of CAC secretome on ECFC angiogenic potential. Tubule formation was measured after incubating ECFC with EBM-2 basal medium (Basal) plus the corresponding factors (50 or 100 ng/ml), ECFC with the 50 or 100 ng/ml of CAC secretome (untreated, control, C50 and C100), or with 50 or 100 ng/ml of secretome of CAC after their incubation with the atherosclerotic factors (AP50, AP100 respectively). Also, a positive control of angiogenesis was tested, incubating ECFC with of 35 mg/mL FGF (activator). GraphPad Prism 7 was used for statistical analysis, using a two-way ANOVA test completed with Tukey-s multiple comparisons test for post hoc analyses.
All comparisons with p-values < 0.05 are shown.

			24h							48h						
			B50	C50	AP50	B100	C100	AP100	FGF	B50	C50	AP50	B100	C100	AP100	FGF
Nb meshes	24h	B50							0.01	<0.001						
		C50							0.004		0.02					
		AP50							<0.001			0.001				
		B100						<0.001					<0.001			
		C100						<0.001						<0.001		
		AP100							<0.001						<0.001	
		FGF														
	48h	B50									<0.001					
		C50										0.02				
		AP50														
		B100														0.04
		C100														
		AP100														
		FGF														
Tot. Meshes area	24h	B50							<0.001	<0.001						
		C50							<0.001							
		AP50						<0.001	<0.001							
		B100											<0.001			
		C100														
		AP100							0.05						<0.001	
		FGF														
	48h	B50									<0.001					<0.001
		C50										<0.001				
		AP50														
		B100												<0.001	0.03	<0.001
		C100														
		AP100														
		FGF														
Nb Segments	24h	B50				<0.001				<0.001						
		C50					<0.001				<0.001					
		AP50							<0.001			<0.001				
		B100						<0.001					<0.001			
		C100						<0.001	<0.001					<0.001		
		AP100							0.02						<0.001	
		FGF														<0.001
	48h	B50														<0.001
		C50														
		AP50														<0.001
		B100												<0.001		<0.001
		C100													<0.001	
		AP100														<0.001
		FGF														
Tot. Segments Lenght	24h	B50			0.04					<0.001						
		C50					<0.001		0.02		<0.001					
		AP50							<0.001			<0.001				
		B100						0.004					<0.001			
		C100						<0.001						<0.001		
		AP100													<0.001	
		FGF														<0.001
	48h	B50									0.004					<0.001
		C50										0.01				
		AP50														<0.001
		B100												<0.001		<0.001
		C100													<0.001	
		AP100														
		FGF														

Supplementary Table S4. Differential proteins identified in the secretome of CAC after incubation *ex vivo* with atherosclerotic factors related to TGFβ1 signaling pathways. According to IPA, TGFβ1 signaling pathway was predicted to be activated, given the number (41 of 67 related proteins) involved in the activation of these pathways

TGFB1

Printed: julio 12, 2020

IPA Build version: exported

ID	Genes in dataset	Prediction (based on measurement direction)	Expr Other	Findings
P18206	VCL	Activated	↑1,000	Upregulates, (3)
P01024	C3	Inhibited	↑1,000	Downregulates, (1)
P36955	SERPINF1	Inhibited	↑1,000	Downregulates, (2)
Q92743	HTRA1	Activated	↑1,000	Upregulates, (2)
P07996	THBS1	Activated	↑1,000	Upregulates, (14)
P35749	MYH11	Activated	↑1,000	Upregulates, (18)
P01871	IGHM	Inhibited	↑1,000	Downregulates, (1)
P24821	TNC	Activated	↑1,000	Upregulates, (18)
Q01995	TAGLN	Activated	↑1,000	Upregulates, (63)
P00734	F2	Inhibited	↑1,000	Downregulates, (1)
P35555	FBN1	Activated	↑1,000	Upregulates, (3)
P04792	HSPB1	Activated	↑1,000	Upregulates, (1)
P08294	SOD3	Activated	↑1,000	Upregulates, (1)
Q9NR12	PDLIM7	Activated	↑1,000	Upregulates, (1)
P35442	THBS2	Activated	↑1,000	Upregulates, (1)
P10909	CLU	Activated	↑1,000	Upregulates, (9)
P13611	VCAN	Activated	↑1,000	Upregulates, (3)
P04003	C4BPA	Activated	↑1,000	Upregulates, (1)
P98160	HSPG2	Activated	↑1,000	Upregulates, (3)
Q05682	CALD1	Activated	↑1,000	Upregulates, (2)
P21980	TGM2	Activated	↑1,000	Upregulates, (16)
P98095	FBLN2	Activated	↑1,000	Upregulates, (4)
P13987	CD59	Activated	↑1,000	Upregulates, (14)
P00751	CFB	Activated	↑1,000	Upregulates, (1)
Q9Y6C2	EMILIN1	Activated	↑1,000	Upregulates, (2)
P12109	COL6A1	Activated	↑1,000	Upregulates, (6)
P68032	ACTC1	Activated	↑1,000	Upregulates, (3)
P30626	SRI	Activated	↑1,000	Upregulates, (1)
P08603	CFH	Activated	↑1,000	Upregulates, (1)
Q99715	COL12A1	Activated	↑1,000	Upregulates, (1)
P02751	FN1	Activated	↑1,000	Upregulates, (139)
Q9UBX5	FBLN5	Activated	↑1,000	Upregulates, (4)
P01042	KNG1	Activated	↑1,000	Upregulates, (1)
P09493	TPM1	Activated	↑1,000	Upregulates, (12)
Q14767	LTBP2	Activated	↑1,000	Upregulates, (2)
P12111	COL6A3	Activated	↑1,000	Upregulates, (4)
P39060	COL18A1	Affected	↑1,000	Regulates, (1)
Q15063	POSTN	Activated	↑1,000	Upregulates, (6)
Q16270	IGFBP7	Activated	↑1,000	Upregulates, (4)
P02649	APOE	Activated	↑1,000	Upregulates, (1)
P21810	BGN	Activated	↑1,000	Upregulates, (19)
Q14766	LTBP1	Activated	↑1,000	Upregulates, (2)
P49411	TUFM	Activated	↓-1,000	Downregulates, (1)
P05107	ITGB2	Inhibited	↓-1,000	Upregulates, (4)
P23368	ME2	Inhibited	↓-1,000	Upregulates, (1)
P06744	GPI	Inhibited	↓-1,000	Upregulates, (1)
O15144	ARPC2	Inhibited	↓-1,000	Upregulates, (1)
P30740	SERPINB1	Inhibited	↓-1,000	Upregulates, (1)
Q9Y3Z3	SAMHD1	Activated	↓-1,000	Downregulates, (1)
P40227	CCT6A	Affected	↓-1,000	Regulates, (2)
P04899	GNAI2	Activated	↓-1,000	Downregulates, (1)
P22897	MRC1	Inhibited	↓-1,000	Upregulates, (1)
P00491	PNP	Affected	↓-1,000	Regulates, (2)
P62191	PSMC1	Inhibited	↓-1,000	Upregulates, (1)
P29350	PTPN6	Inhibited	↓-1,000	Upregulates, (1)
P07900	HSP90AA1	Inhibited	↓-1,000	Upregulates, (1)
P48643	CCT5	Inhibited	↓-1,000	Upregulates, (3)
P31949	S100A11	Inhibited	↓-1,000	Upregulates, (2)
Q9NRV9	HEBP1	Affected	↓-1,000	Regulates, (1)
P02730	SLC4A1	Inhibited	↓-1,000	Upregulates, (1)
P48506	GCLC	Activated	↓-1,000	Downregulates, (8)
P63244	RACK1	Inhibited	↓-1,000	Upregulates, (5)
P09601	HMOX1	Inhibited	↓-1,000	Upregulates, (19)
P43490	NAMPT	Affected	↓-1,000	Regulates, (1)
P08575	PTPRC	Affected	↓-1,000	Regulates, (1)
P63000	RAC1	Inhibited	↓-1,000	Upregulates, (1)
Q09666	AHNAK	Inhibited	↓-1,000	Upregulates, (3)

Supplementary Table S5.

Primary antibodies used in this study

Antibody	Reactivity	Dilution	Suplier	Reference	Used in
CD31-FITC	H	1:25	Biolegend	303103	FC
CD34-APC	H	1:25	Biolegend	343607	FC
CD45-PBlue	H	1:25	Biolegend	368539	FC
CD90-PE	H	1:25	Biolegend	328109	FC
CD73-FITC	H	1:25	Biolegend	344015	FC
CD105-FITC	H	1:25	Biolegend	323203	FC
CD309-PE	H	1:25	Biolegend	359903	FC
CD133-PE	H	1:25	Miltenyi Biotec	130-098-826	FC
CD146-PE	H	1:25	Biolegend	361005	FC
CD14-FITC	H	1:25	Biolegend	367115	FC
Rabbit-anti-THBS1	H, R, M	1:500	Proteintech	18304-1-AP	WB
Goat-anti-Apo-E	H	1:3000	Sigma-Aldrich	SAB2500086	WB

Secondary antibodies used in this study

Antibody	Reactivity	Dilution	Suplier	Reference	Used in
HRP anti-rabbit IgG	R	1:5000	Novus	NB7160	WB
HRP anti-goat IgG	G	1:2000	Thermo Fisher	61-1620	WB

FC: Flow cytometry; **WB**: Western Blot; **H**: Human; **R**: Rat; **M**:mouse; **G**: Goat

Additionally, for cell detection FITC-UEA-1 (1:300, Sigma L9006) and DiI-ac-LDL (Biomedical Technologies, BT-902, Stoughton, MA, USA) were used.
